# Field-Orientation Effects in Amplitude-Modulation Magnetic Particle Imaging for High-Resolution Navigation

**DOI:** 10.3390/s26175420

**Published:** 2026-08-27

**Authors:** Loc Phuoc Nguyen, Tuan-Anh Le, Muhammad Auwal Shehu, Yussuf Shakhin, Ton Duc Do

**Affiliations:** 1 School of Computing and Artificial Intelligence (SCAI), Nazarbayev University, Astana 010000, Kazakhstan; loc.np@nu.edu.kz (L.P.N.); auwal.shehu@nu.edu.kz (M.A.S.); yussuf.shakhin@nu.edu.kz (Y.S.); 2 Medical Microrobots Lab, Department of Physiology and Biomedical Engineering, Mayo Clinic, Scottsdale, AZ 85259, USA; le.tuananh@mayo.edu

**Keywords:** electromagnetic sensors, magnetic particle imaging, field-free point, field-free line, spatial resolution, nanorobots

## Abstract

Amplitude-modulation magnetic particle imaging (AM-MPI) has strong potential for real-time navigation of magnetic nanoparticles because it provides tracer-specific spatial feedback using a narrowband acquisition scheme with relatively low excitation power and simplified signal detection. However, improving spatial resolution by increasing the selection-field gradient becomes increasingly difficult as scanner dimensions increase. This study investigates whether field orientation can be used to improve and control the spatial resolution of three-dimensional AM-MPI with field-free-point (FFP) and field-free-line (FFL) encoding. Four scan-receive configurations were analyzed using a matrix point-spread-function (PSF) model, followed by two-point phantom simulations at equal physical and FWHM-normalized source separations. With a common y-directed scan, FFP-y produced a single-peaked collinear *h_yy_* response with FWHM values of 1.29 mm along y and 5.89 mm along x and z. FFL-y retained the same y-direction FWHM while reducing the z-direction FWHM to 2.94 mm. FFP-x selected the transverse *h_xy_* component, producing a central null and a multi-lobe response, whereas FFL-x was a null channel for the adopted field geometry. At equal physical spacing, the first tested separation satisfying the adopted Rayleigh-type criterion (*M* ≥ 0.26) was 3 mm along *y* for both FFP-y and FFL-y, 5 mm along *z* for FFL-y, and 7 mm along *x* and *z* for FFP-y. FFL-y also provided better z-direction separability than FFP-y. After normalization by the corresponding directional FWHM, the single-peaked responses showed similar two-point separability, indicating that the differences observed at equal physical spacing were mainly associated with directional PSF width. These results show that field orientation affects both the topology and directional resolution of the AM-MPI response and can be considered together with selection-field design when optimizing AM-MPI systems for nanoparticle navigation.

## 1. Introduction

The navigation of micro- and nanoscale therapeutic agents has garnered significant attention for applications such as targeted drug delivery, localized therapy, microrobot guidance, and minimally invasive interventions [1,2,3]. Irrespective of the propulsion mechanism—magnetic, mechanical, acoustic, or hybrid—effective navigation necessitates spatial feedback to determine the location and distribution of therapeutic agents relative to the intended target. Conventional imaging modalities, including magnetic resonance imaging (MRI), computed tomography (CT), positron emission tomography (PET), and ultrasound (US), offer valuable anatomical and functional insights [4,5,6]. Nevertheless, their application in nanoparticle navigation is often constrained by factors such as ionizing radiation, system complexity, limited accessibility, insufficient tracer sensitivity, or inadequate suitability for continuous position feedback [7,8,9,10].

Magnetic particle imaging (MPI) is a tracer-specific modality that directly detects the nonlinear magnetization response of magnetic nanoparticles (MNPs), particularly superparamagnetic iron oxide nanoparticles [11,12,13,14]. As MPI generates a signal directly from the tracer rather than from surrounding tissue, it offers high contrast, high sensitivity, and radiation-free detection of MNP distributions [15,16]. These attributes render MPI highly suitable for navigation systems utilizing MNPs as therapeutic carriers, microrobot components, or detectable labels [17,18]. In this context, MPI provides spatial feedback independently of the actuation mechanism, thereby supporting magnetic, nonmagnetic, or hybrid navigation strategies.

**Figure 1:**
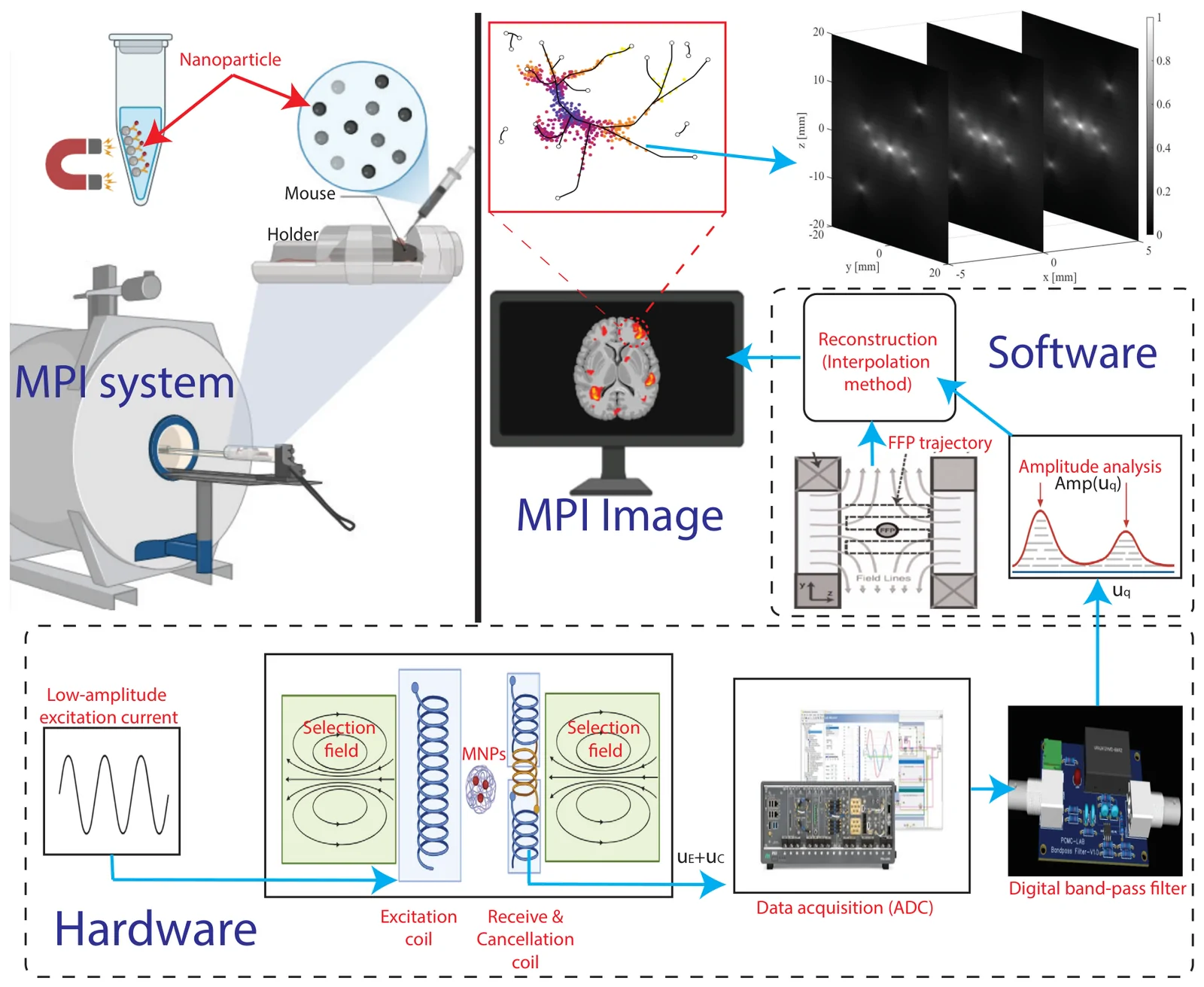
Overview of the AM-MPI system for position feedback through FFP/FFL scanning of magnetic nanoparticles.

AM-MPI has been established as a real-time method for localizing MNPs during navigation and targeted drug delivery [19,20]. In contrast to conventional broadband MPI approaches, which reconstruct particle distributions from multiple nonlinear harmonic components, AM-MPI employs a low-amplitude, high-frequency excitation field and detects the amplitude envelope of the resulting narrowband particle signal [21,22]. Following suppression of direct excitation feedthrough, the carrier amplitude is extracted and mapped to the instantaneous FFP or FFL position. This narrowband acquisition approach reduces excitation-power requirements, simplifies signal detection, and has facilitated the development of AM-MPI systems ranging from small-scale platforms to human-head-scale systems [23,24,25]. The overall AM-MPI hardware, signal-acquisition, and reconstruction workflow is summarized in Figure 1.

However, scaling AM-MPI to larger navigation systems introduces significant hardware constraints. Achieving stronger selection gradients over an expanded field of view typically necessitates higher coil currents, increased electrical power, more rigorous thermal management, and coil configurations that may limit bore accessibility or reduce the available distance between the imaging volume and the field-generating coils [26]. These limitations become more pronounced as system dimensions and imaging volume increase. As a result, spatial resolution cannot always be enhanced solely by increasing the selection-gradient strength.

Spatial resolution is a critical parameter in navigation-oriented MPI, as it determines the accuracy of MNPs localization, the ability to distinguish closely spaced targets and particle accumulations, and the reliability of position feedback for closed-loop guidance [27]. A broad or highly anisotropic spatial resolution can blur the estimated tracer distribution, introduce direction-dependent localization errors, and diminish the precision with which a therapeutic carrier can be guided toward a target. Consequently, enhancing spatial resolution through field-configuration design is a key engineering objective, especially when further increases in selection-gradient strength are limited by the physical, electrical, and thermal constraints of large-scale systems.

Spatial resolution in AM-MPI depends on tracer properties, selection-gradient strength, scanning conditions, receive-coil sensitivity, and signal processing. The relative orientation of the selection, scan, excitation, and receive fields has received less attention, although it can directly change the spatial response measured by the receive channel. In many existing AM-MPI systems, field and coil orientations have primarily been determined by bore geometry, coil accessibility, inductive decoupling, and excitation-feedthrough suppression requirements [28,29]. A frequently adopted configuration places the excitation and receive axes perpendicular to the principal selection-gradient direction. While this arrangement is practical for hardware implementation, it may not yield the most favorable spatial response for navigation.

In the equilibrium x-space description, the spatial resolution is represented by a matrix-valued PSF rather than by a single scalar susceptibility profile. The matrix PSF is determined by the selection-gradient tensor and the Langevin magnetization law. The measured scalar channel is obtained by projecting this matrix onto the receive-sensitivity direction and the instantaneous FFP/FFL translation direction. The high-frequency AM excitation generates the narrowband carrier used for detection, but it does not replace the scan-direction vector in this spatial PSF projection. The collinear component is measured when the fast scan is performed along the *y* axis and the receiver is aligned with the same axis. In contrast, the transverse component is measured when the fast scan is performed along the *y* axis while the receiver is aligned with the *x* axis. This distinction is essential because the transverse PSF can exhibit sign changes, multiple lobes, and a central null even under otherwise identical tracer and gradient conditions [30].

The influence of field orientation on spatial resolution in three-dimensional AM-MPI remains insufficiently characterized. With FFP encoding, the anisotropic selection gradient makes the measured response sensitive to the relative orientation of the scan and receive axes; a configuration that produces a narrow response in one direction may broaden or alter the response in another. FFL encoding introduces an additional constraint because the selection gradient is zero along the field-free line. The response is, therefore, localized only in the encoded transverse plane and remains nonlocal along the line unless supplementary encoding is introduced. A comparison of FFP and FFL configurations must, therefore, separate changes associated with scan-receive orientation from those imposed by the field-free geometry itself.

This study examines the effect of field orientation on the spatial resolution of three-dimensional AM-MPI using the same tracer properties, principal y-gradient magnitude, excitation conditions, and y-directed scan. Four FFP and FFL scan-receive configurations are compared within a common matrix-PSF framework. With the scan fixed along y, FFP-y and FFP-x select the collinear $h_{yy}$ and transverse $h_{xy}$ components, respectively, while the FFL cases distinguish the encoded transverse response from the null-gradient line direction. Spatial performance is evaluated according to response topology: conventional FWHM is used for single-peaked collinear profiles, whereas the transverse multi-lobe response is characterized using lobe-specific measures and the null channel is not assigned a spatial width. Two-point phantom simulations are then used to compare source separability at equal physical and FWHM-normalized spacings. This approach separates the practical effect of directional PSF width from the localization properties imposed by FFP and FFL geometry.

## 2. Principles and Problem Formulation

### 2.1. AM-MPI Imaging Principle and Field Configurations

**Figure 2:**
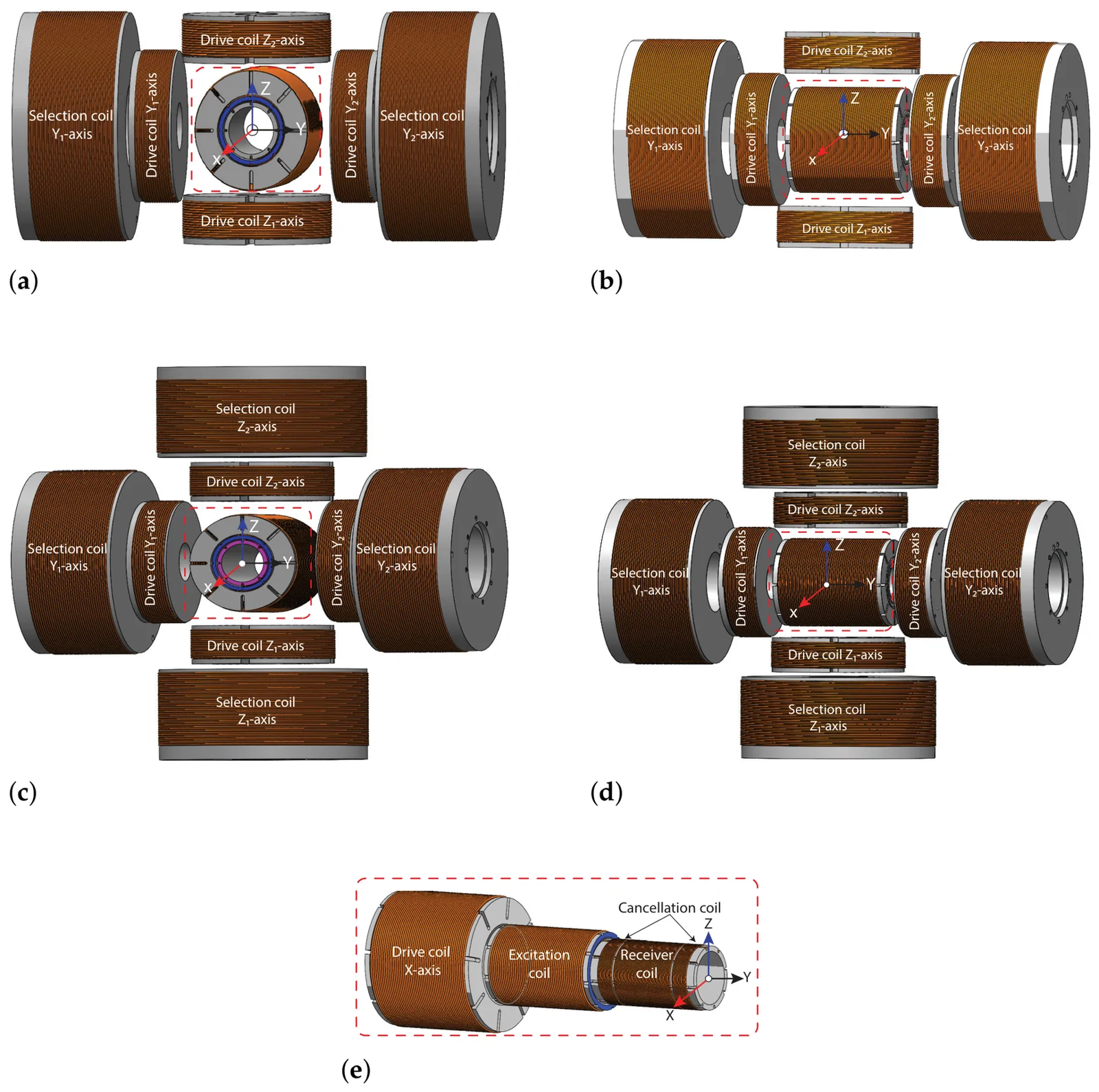
Coil topologies of the investigated AM-MPI field-orientation configurations. (**a**) FFP-*x*-excitation configuration, transverse to the principal *y*-gradient. (**b**) FFP-*y*-excitation configuration, aligned with the principal *y*-gradient. (**c**) FFL-*y*-excitation configuration, with excitation and receive axes transverse to the *x*-directed FFL. (**d**) FFL-*x*-excitation configuration, with excitation and receive axes parallel to the *x*-directed FFL. (**e**) Drive, excitation, receive, and cancellation coil arrangement.

This work considers an AM-MPI system based on the principles outlined in Section 1. The system includes several key components: a selection-gradient coil system, drive coils, an excitation coil, a receive coil, and a cancellation coil, as shown in Figure 2. The selection-gradient field produces either an FFP or an FFL, which defines the spatial region to which the scanner is sensitive. Low-frequency drive fields then translate the FFP or FFL across the field of view. At the same time, a low-amplitude, high-frequency excitation field induces an oscillatory response in MNPs. The receive coil picks up the voltage generated by these particles. The cancellation coil reduces interference from the excitation feedthrough prior to band-pass filtering and amplitude-envelope extraction.

In this setup, the selection-gradient field outlines the spatial encoding geometry, the drive field shapes the scanning path, and the alignment of the excitation and receive axes determines which components of susceptibility are measured. Because of this, the orientation of the excitation-receive axis relative to the selection-gradient field significantly affects the demodulated amplitude response, the PSF, and the overall spatial resolution. This study compares four AM-MPI field-orientation configurations: FFP-*x*-excitation, FFP-*y*-excitation, FFL-*y*-excitation, and FFL-*x*-excitation. For the FFP geometry, the labels distinguish excitation transverse to and aligned with the principal *y*-gradient, respectively. For the *x*-directed FFL geometry, FFL-*y*-excitation denotes excitation along an encoded transverse direction, whereas FFL-*x*-excitation denotes excitation parallel to the FFL. In all four configurations, the receive coil is assumed to be collinear with the excitation coil.

Due to the symmetry of the adopted selection-gradient tensors, excitation along the *z*-axis does not provide an independent field-orientation configuration. For the FFP geometry, the transverse gradients satisfy $G_{x}=G_{z}=-\frac{G}{2}$; therefore, excitation along *z* is symmetrically equivalent to the investigated FFP-*x*-excitation case under interchange of the *x*- and *z*-coordinates. For the FFL geometry, the encoded transverse gradients satisfy $G_{y}=-G_{z}$, and therefore, have equal magnitude. The *y*-direction is selected as the representative transverse excitation and fast-translation direction for the FFL projection scan, while the *z*-direction is used for the slower orthogonal raster update.

It is worth noting that the focus field is not included in the present analysis. Although a focus field can shift the location of the field-free region or extend the field of view in a typical MPI scanner, the primary objective of this study is to investigate the effects of local field orientation under consistent selection-gradient and drive-field conditions. Excluding the focus field allows the influence of the relative alignment between the excitation and receive directions on the PSF and FWHM to be examined more clearly. Accordingly, the total magnetic field acting on a particle is modeled solely as the superposition of the selection-gradient field, the low-frequency drive field, and the high-frequency excitation field.

The total magnetic field experienced by a particle at position $r={\left[x,y,z\right]}^{T}$ and time *t* can be written as

(1) $$H\left(r,t\right)=H_{S}\left(r\right)+H_{D}\left(t\right)+H_{E}\left(t\right)$$

where $H_{S}\left(r\right)=Gr$ is the total selection-gradient field forming the FFP or FFL. The low-frequency drive field generates the prescribed line-by-line trajectory, while the high-frequency excitation field generates the AM-MPI carrier response. The fast translation is fixed along the *y*-direction for all four configurations; only the slower raster dimensions differ between the fully-localized FFP and the transversely-encoded FFL geometry. Therefore, $H_{D}\left(t\right)$ is retained in vector form and specified by the geometry-dependent trajectory below.

Because the FFP and FFL selection-gradient matrices have different mathematical properties, their trajectories are formulated separately. For the FFP configuration, the selection-gradient matrix is defined as

(2) $$G_{\mathrm{FFP}}=\left[\begin{array}{ccc} -\frac{G}{2} & 0 & 0\\ 0 & G & 0\\ 0 & 0 & -\frac{G}{2} \end{array}\right],$$

which satisfies the Maxwell condition

(3) $$G_{x}+G_{y}+G_{z}=0.$$

Because $G_{\mathrm{FFP}}$ is nonsingular, the instantaneous FFP position is defined only by the slowly varying selection and drive fields:

(4) $$H_{S,\mathrm{FFP}}\;\left(r_{\mathrm{FFP}}\left(t\right)\right)+H_{D,\mathrm{FFP}}\left(t\right)=0.$$

where the selection field is given by

(5) $$H_{S,\mathrm{FFP}}\left(r\right)=G_{\mathrm{FFP}}r,$$

and the drive-defined FFP trajectory is

(6) $$r_{\mathrm{FFP}}\left(t\right)=-G_{\mathrm{FFP}}^{-1}H_{D,\mathrm{FFP}}\left(t\right).$$

The high-frequency excitation field is not included in the FFP trajectory definition; instead, it is treated as a small perturbation used to probe the local differential susceptibility. Therefore, the total field can be written as

(7) $$H_{\mathrm{FFP}}\left(r,t\right)=G_{\mathrm{FFP}}\left(r-r_{\mathrm{FFP}}\left(t\right)\right)+H_{E}\left(t\right).$$

This formulation distinguishes the low-frequency spatial scanning trajectory from the high-frequency excitation perturbation. Its validity is due to the fact that the FFP is localized in all three spatial directions.

For the FFL configuration, the selection-gradient matrix is defined as

(8) $$G_{\mathrm{FFL}}=\left[\begin{array}{ccc} 0 & 0 & 0\\ 0 & G & 0\\ 0 & 0 & -G \end{array}\right],$$

which satisfies

(9) $$G_{x}=0,\;G_{y}=-G_{z}.$$

In this case, the y- and z- directions constitute symmetry-equivalent transverse encoding directions in terms of gradient magnitude. Thus, the field-free region is a line extending along the *x*-axis. Since the first row of $G_{\mathrm{FFL}}$ is zero, the matrix is singular and a full three-dimensional inverse $G_{\mathrm{FFL}}^{-1}$ does not exist. Therefore, the FFL position is described only in the transverse encoded plane.

The transverse FFL position is obtained from the zero-field condition in the *y*- and *z*-directions:

(10) $$G_{y}y_{\mathrm{FFL}}\left(t\right)+H_{D_{y}}\left(t\right)=0,$$

(11) $$G_{z}z_{\mathrm{FFL}}\left(t\right)+H_{D_{z}}\left(t\right)=0.$$

Therefore,

(12) $$y_{\mathrm{FFL}}\left(t\right)=-\frac{H_{D_{y}}\left(t\right)}{G_{y}},\;z_{\mathrm{FFL}}\left(t\right)=-\frac{H_{D_{z}}\left(t\right)}{G_{z}}.$$

Equivalently, the transverse FFL trajectory can be written as

(13) $$r_{\mathrm{FFL},⊥}\left(t\right)=\left[\begin{array}{c} y_{\mathrm{FFL}}\left(t\right)\\ z_{\mathrm{FFL}}\left(t\right) \end{array}\right]=-{\left[\begin{array}{cc} G_{y} & 0\\ 0 & G_{z} \end{array}\right]}^{-1}\left[\begin{array}{c} H_{D_{y}}\left(t\right)\\ H_{D_{z}}\left(t\right) \end{array}\right].$$

The complete FFL can then be represented as

(14) $$r_{\mathrm{FFL}}\left(s,t\right)=\left[\begin{array}{c} s\\ y_{\mathrm{FFL}}\left(t\right)\\ z_{\mathrm{FFL}}\left(t\right) \end{array}\right],\;s\in R,$$

where *s* is the free coordinate along the FFL direction. Because $G_{x}=0$, a drive-field component along the FFL cannot be spatially canceled. Therefore, the FFL simulations use

(15) $$H_{D_{x}}=0,$$

and the line is translated only in the encoded transverse *y*-*z* plane. The corresponding magnetic field is

(16) $$H_{\mathrm{FFL}}\left(r,t\right)=G_{\mathrm{FFL}}r+H_{D_{y}}\left(t\right){\hat{e}}_{y}+H_{D_{z}}\left(t\right){\hat{e}}_{z}+H_{E}\left(t\right).$$

This formulation explicitly shows that FFL-based encoding is local only in the transverse *y*-*z* plane and remains nonlocal along the *x*-direction unless supplementary spatial encoding is introduced.

The spatial comparison is performed with a common fast translation along the *y*-direction. The excitation and receive coils are rotated together for the hardware configurations, but the spatial PSF projection must distinguish the receive direction from the translation direction. Let ${\hat{p}}_{q}$ denote the unit receive-sensitivity direction and ${\hat{v}}_{F,q}$ the unit translation direction of the field-free region. The investigated receive directions are

(17) $${\hat{p}}_{q}=\left\{\begin{array}{cc} {\hat{e}}_{x}, & q\in \{\mathrm{FFP}\text{-}x,\mathrm{FFL}\text{-}x\},\\ {\hat{e}}_{y}, & q\in \{\mathrm{FFP}\text{-}y,\mathrm{FFL}\text{-}y\}, \end{array}\right.$$

whereas the common fast-scan direction is

(18) $${\hat{v}}_{F,q}={\hat{e}}_{y},\;q\in \left\{\mathrm{FFP}\text{-}x,\mathrm{FFP}\text{-}y,\mathrm{FFL}\text{-}x,\mathrm{FFL}\text{-}y\right\}.$$

Following the multidimensional x-space formulation, the matrix PSF h(r) itself does not depend on the choice of the FFP velocity unit vector; however, the scalar receive channel does depend on which matrix component is selected, namely

(19) $$h_{q}\left(r\right)={\hat{p}}_{q}^{T}h\left(r\right){\hat{v}}_{F,q}.$$

Thus, for the FFP with a common *y*-directed scan, FFP-*y* reception selects $h_{yy}={\hat{e}}_{y}^{T}h{\hat{e}}_{y}$ and is collinear, whereas FFP-*x* reception selects $h_{xy}={\hat{e}}_{x}^{T}h{\hat{e}}_{y}$ and is transverse. This is exactly the same projection principle used for the collinear and transverse components in multidimensional x-space MPI [30]. The approximately 370% collinear-to-transverse peak-amplitude difference reported in that work is therefore relevant when considering amplitude and SNR, although the exact ratio is not imposed as a numerical constraint in the present simulations.

The same projection rule is applied to the FFL. Because the adopted FFL is directed along *x* and has $G_{x}=0$, the ideal matrix-PSF channel with receive direction *x* and translation direction *y* is null. A nonzero transverse FFL comparison under the common *y*-scan must instead use a receive direction within the encoded *y*–*z* plane, for example, *z*. The consequences of this property are derived explicitly in Section 3.3.

### 2.2. FFP/FFL Generation and Line-by-Line Scanning

**Figure 3:**
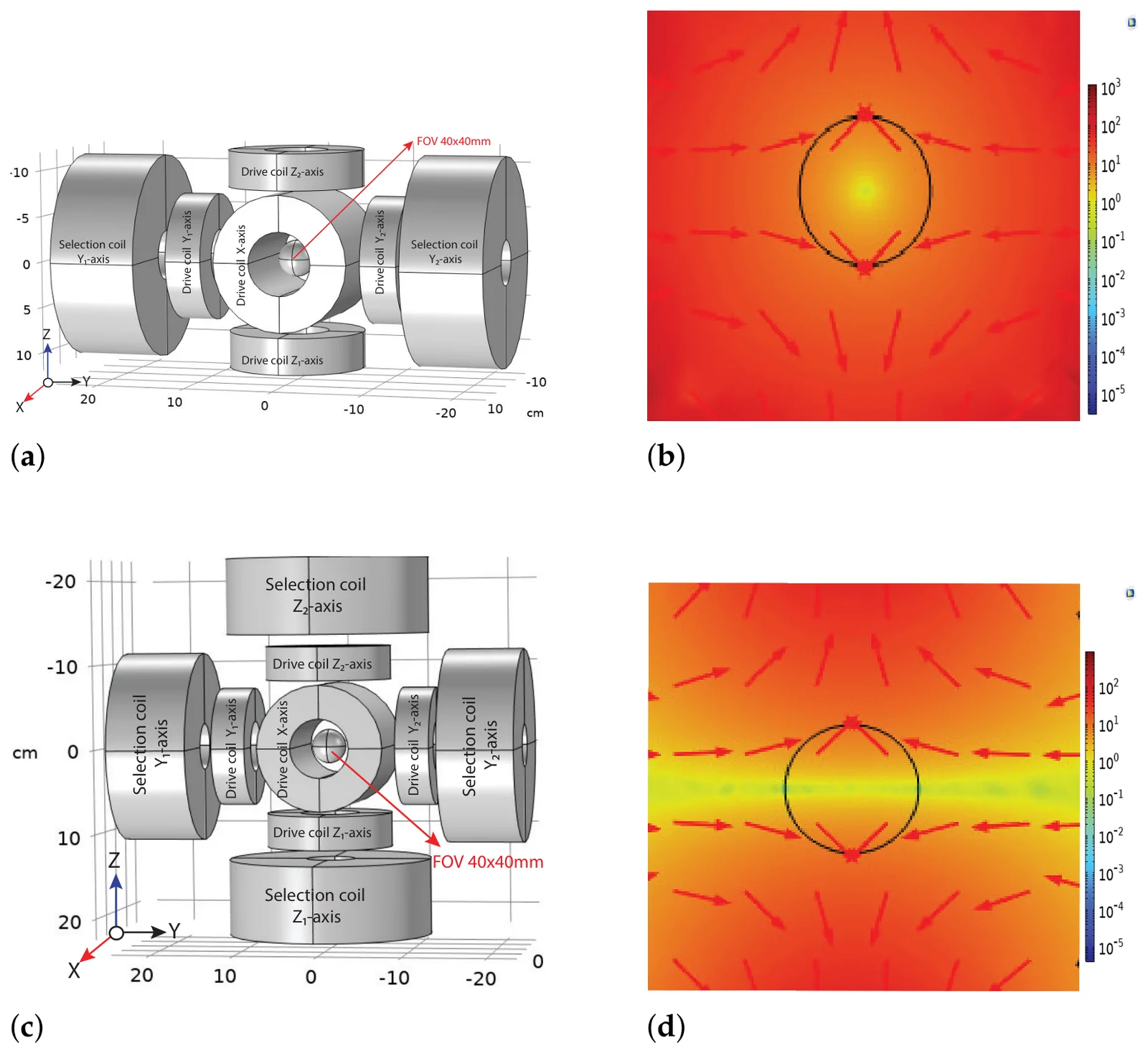

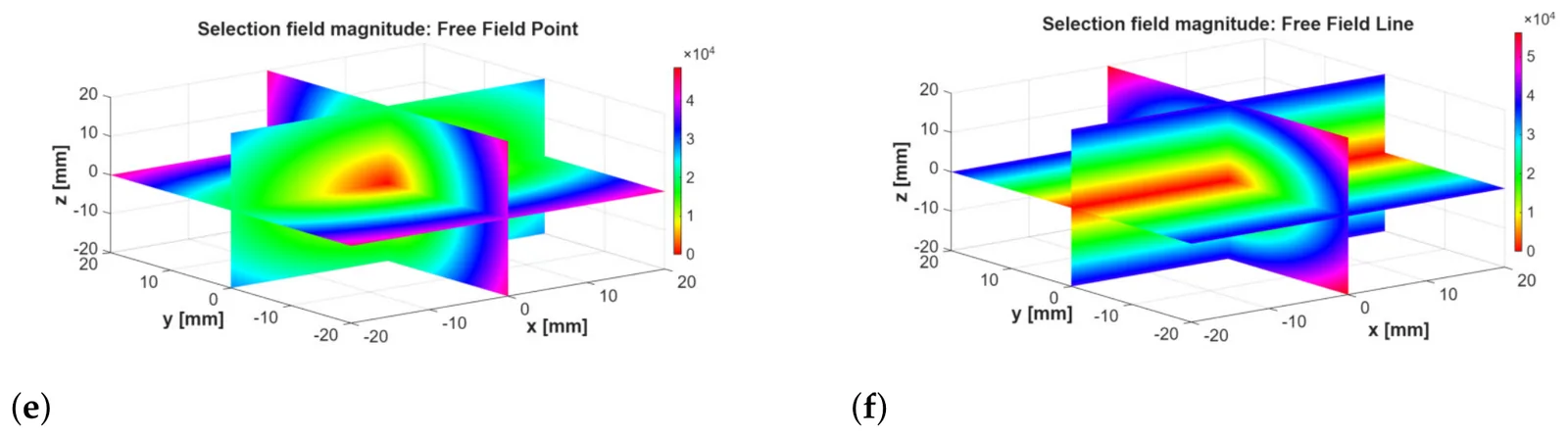
Field-free-region generation and selection-field distributions for FFP- and FFL-based AM-MPI spatial encoding. (**a**,**b**) COMSOL (version 6.2, AC/DC Module) model and selection-field distribution of the FFP configuration. (**c**,**d**) COMSOL model and selection-field distribution of the FFL configuration. (**e**,**f**) Three-dimensional selection-field distributions of the FFP and FFL configurations, respectively, simulated using MATLAB R2025b (The MathWorks, Inc.).

The selection-gradient field defines the FFP or FFL geometry, whereas the low-frequency drive fields translate the field-free region through the field of view. To isolate orientation effects from trajectory effects, the fast translation is fixed along the same *y*-direction for all four configurations. The corresponding reconstructed profiles for the investigated field orientations are presented in Figure 3.

*FFP scanning:* For FFP imaging, the field-free point is localized in all three spatial directions. Both FFP-*x*-excitation and FFP-*y*-excitation, therefore, use the same line-by-line raster geometry, with the fast coordinate along *y*. The trajectory is

(20) $$r_{\mathrm{scan},\mathrm{FFP}}\left(\tau ,n,m\right)=r_{f}\left(\tau \right){\hat{e}}_{y}+r_{l}\left(n\right){\hat{e}}_{x}+r_{s}\left(m\right){\hat{e}}_{z},$$

where τ denotes the local time within a fast line, *n* is the line index, and *m* is the slice index. The corresponding drive field is

(21) $$H_{D,\mathrm{FFP}}\left(\tau ,n,m\right)=-G_{\mathrm{FFP}}r_{\mathrm{scan},\mathrm{FFP}}\left(\tau ,n,m\right).$$

The two FFP orientation cases are, therefore,

(22) $$\begin{array}{cc} \mathrm{FFP}\text{-}x\text{-}\mathrm{excitation}: & \;{\hat{e}}_{f}={\hat{e}}_{y},\;u_{q}={\hat{p}}_{q}={\hat{e}}_{x}, \end{array}$$

(23) $$\begin{array}{cc} \mathrm{FFP}\text{-}y\text{-}\mathrm{excitation}: & \;{\hat{e}}_{f}={\hat{e}}_{y},\;u_{q}={\hat{p}}_{q}={\hat{e}}_{y}. \end{array}$$

Thus, with the common *y*-directed translation, the FFP-*x* hardware configuration selects the transverse matrix-PSF component $h_{xy}$, whereas the FFP-*y* configuration selects the collinear component $h_{yy}$. The phantom position or orientation does not alter this scanner-defined assignment.

*FFL scanning:* The FFL geometry is treated differently because $G_{\mathrm{FFL}}$ is singular. The field-free line extends along *x*, and $G_{x}=0$. Therefore,

(24) $$H_{D_{x}}=0,$$

and all spatial translation is restricted to the encoded *y*–*z* plane. For both FFL excitation–receive orientations, the same fast translation along *y* and a common slower orthogonal update along *z* are used:

(25) $$r_{\mathrm{scan},\mathrm{FFL},⊥}\left(\tau ,m\right)=r_{f}\left(\tau \right){\hat{e}}_{y}+r_{s}\left(m\right){\hat{e}}_{z}.$$

The required drive fields are obtained from the transverse zero-field condition,

(26) $$\left[\begin{array}{c} H_{D_{y}}\left(\tau \right)\\ H_{D_{z}}\left(m\right) \end{array}\right]=-\left[\begin{array}{cc} G_{y} & 0\\ 0 & G_{z} \end{array}\right]\left[\begin{array}{c} r_{f}\left(\tau \right)\\ r_{s}\left(m\right) \end{array}\right].$$

The two FFL orientation cases are

(27) $$\begin{array}{c} \mathrm{FFL}\text{-}x\text{-}\mathrm{excitation}:\;u_{q}={\hat{p}}_{q}={\hat{e}}_{x}, \end{array}$$

(28) $$\begin{array}{c} \mathrm{FFL}\text{-}y\text{-}\mathrm{excitation}:\;u_{q}={\hat{p}}_{q}={\hat{e}}_{y}. \end{array}$$

Accordingly, FFL-*y* reception is collinear with the fast translation in the encoded plane. FFL-*x* reception is parallel to the *x*-directed field-free line; because $G_{x}=0$, this direction is a null channel of the ideal matrix-PSF translation model rather than a nonzero transverse spatial response. The designation FFL-*x* refers to the hardware sensing orientation and does not imply an *x*-directed spatial scan.

The common *y*-directed fast scan provides the controlled structure used throughout this study: within each field-free geometry, the selection-gradient tensor and scan trajectory are held fixed, while only the excitation–receive sensing orientation is changed. This avoids conflating an orientation-dependent change in the projected magnetic susceptibility with a simultaneous change in the raster trajectory.

(i) FFP configuration: $G_{y}=-2G_{x}=-2G_{z}$. The field-free region is localized in three dimensions. Both FFP cases scan rapidly along *y*; the receive/excitation direction is *x* or *y*.
(ii) FFL configuration: $G_{x}=0$ and $G_{y}=-G_{z}$. The field-free region is an *x*-directed line. Both FFL cases scan rapidly along *y* in the encoded transverse plane; the receive/excitation direction is *x* or *y*.

## 3. Methodology

### 3.1. Orientation-Dependent AM-MPI Signal Model

The AM-MPI signal model separates the high-frequency excitation from the low-frequency field-free-region translation. The high-frequency excitation generates the carrier signal, while the low-frequency translation provides spatial encoding. For each configuration (*q*), the total field is

(29) $$H_{q}\left(r,t\right)=H_{0,q}\left(r,t\right)+H_{E,q}\left(t\right),$$

where

(30) $$H_{0,q}\left(r,t\right)=H_{S,q}\left(r\right)+H_{D,q}\left(t\right),$$

and

(31) $$H_{E,q}\left(t\right)=H_{E}\sin\left(2\pi f_{E}t\right)u_{q}.$$

Here, $H_{E}=2\;\mathrm{mT}/\mu_{0}$ and $f_{E}=20\;\mathrm{kHz}$. The excitation direction $u_{q}$ is collinear with the receive coil in the investigated hardware configurations,

(32) $$\begin{array}{cc} u_{\mathrm{FFP}\text{-}x}={\hat{e}}_{x},\;\;\;\;\;\;\;\; & u_{\mathrm{FFP}\text{-}y}={\hat{e}}_{y}, \end{array}$$

(33) $$\begin{array}{cc} u_{\mathrm{FFL}\text{-}x}={\hat{e}}_{x},\;\;\;\;\;\;\;\; & u_{\mathrm{FFL}\text{-}y}={\hat{e}}_{y}, \end{array}$$

and the receive sensitivity is approximated as spatially uniform,

(34) $$p_{q}\left(r\right)=p_{0}{\hat{p}}_{q}.$$

For an equilibrium monodisperse Langevin model in [31], the MNPs magnetization is

(35) $$M_{q}\left(r,t\right)=mc\left(r\right)L\;\left(\beta ∥H_{q}\left(r,t\right)∥\right)\frac{H_{q}\left(r,t\right)}{∥H_{q}\left(r,t\right)∥},$$

where $\beta =\mu_{0}m/\left(k_{B}T\right)$. Brownian relaxation, Néel relaxation, dynamic phase lag, and particle-size distributions are not considered in the present model.

Linearization about the slowly varying field $H_{0,q}$ gives the differential susceptibility tensor

(36) $$\chi_{q}=\chi_{⊥,q}\left(I-{\hat{h}}_{q}{\hat{h}}_{q}^{T}\right)+\chi_{\|,q}{\hat{h}}_{q}{\hat{h}}_{q}^{T},$$

with

(37) $$\chi_{\|,q}=\beta L^{\prime }\left(\beta h_{q}\right),\;\chi_{⊥,q}=\frac{L(\beta h_{q})}{h_{q}},\;h_{q}=∥H_{0,q}∥.$$

For spatial encoding, however, the relevant quantity is not the diagonal excitation projection ${\hat{p}}^{T}\chi u_{q}$ alone. Translation of the FFP/FFL through the selection field introduces the gradient tensor. The matrix-valued spatial point-spread function (PSF) is expressed in terms of the differential susceptibility tensor as

(38) $$h\left(\Delta r\right)=\frac{1}{\beta }\chi \left(\Delta r\right)G$$

### 3.2. Narrowband Demodulation and Envelope Extraction

The voltage measured by the receive coil contains the particle response and direct excitation feedthrough,

(39) $$u\left(t\right)=u^{PR}\left(t\right)+u^{E}\left(t\right).$$

A cancellation coil suppresses the directly induced component so that, ideally,

(40) $$u\left(t\right)=u^{PR}\left(t\right)+u^{E}\left(t\right)+u^{C}\left(t\right)\approx u^{PR}\left(t\right),\;u^{C}\left(t\right)\approx -u^{E}\left(t\right).$$

The scale separation between the low-frequency raster scan and the high-frequency excitation also clarifies which temporal-derivative terms contribute to the AM-MPI carrier. Writing the magnetization as

(41) $$M_{q}\approx M_{0,q}\left(H_{0,q}\right)+mc\left(r\right)\chi_{q}\left(H_{0,q}\right)u_{q}H_{E}\sin\left(\omega_{E}t\right),\;\omega_{E}=2\pi f_{E},$$

its derivative contains

(42) $$\begin{array}{cc} \frac{dM_{q}}{dt}\approx & \;\frac{dM_{0,q}}{dt}+mc\left(r\right)H_{E}\chi_{q}u_{q}\omega_{E}\cos\left(\omega_{E}t\right)\\ \; & +mc\left(r\right)H_{E}\frac{d\chi_{q}}{dt}u_{q}\sin\left(\omega_{E}t\right). \end{array}$$

The first term is associated with the slowly varying drive-field scan and lies predominantly at the scan-rate frequencies. The second term is the dominant carrier-frequency response generated by the high-frequency excitation. The third term represents slow modulation of the susceptibility during the scan. If $f_{D,\max}$ denotes the highest characteristic scan-frequency, then

(43) $$f_{D,\max}\ll f_{E},$$

and the magnitude of the susceptibility-derivative contribution relative to the dominant carrier term scales as

(44) $$\frac{∥{\dot{\chi }}_{q}∥}{\omega_{E}∥\chi_{q}∥}=O\;\left(\frac{f_{D,\max}}{f_{E}}\right)\ll 1.$$

Accordingly, the slowly varying susceptibility-derivative term is neglected in the present idealized AM-MPI model. The band-pass stage additionally rejects the low-frequency scan-induced contribution from the equilibrium magnetization, while retaining the carrier around the excitation frequency.

The residual particle signal is processed with a fifth-order Butterworth digital band-pass filter over 15–25 kHz. The image-forming quantity is the extracted carrier envelope,

(45) $$A_{q}\;\left(r_{F}\left(t\right)\right)=\mathrm{Amp}\;\left[u^{PR}\left(t\right)\right].$$

### 3.3. Point-Spread Function Model for FFP and FFL AM-MPI

Following the multidimensional x-space formulation, the AM-MPI spatial PSF is represented by a matrix. Let

(46) $$\Delta r={\left[\Delta x,\Delta y,\Delta z\right]}^{T},\;H=∥G\Delta r∥,\;n=\frac{G\Delta r}{H}.$$

The matrix PSF is

(47) $$h\left(\Delta r\right)=L^{\prime }\left(\beta H\right)\;nn^{T}G+\frac{L(\beta H)}{\beta H}\left(I-nn^{T}\right)G.$$

To simplify the notation, we define

(48) $$A\left(H\right)=L^{\prime }\left(\beta H\right),\;B\left(H\right)=\frac{L(\beta H)}{\beta H}.$$

Then

(49) $$h\left(\Delta r\right)=BG+\left(A-B\right)nn^{T}G.$$

#### 3.3.1. FFP Collinear and Transverse Components

For the FFP,

(50) $$G_{\mathrm{FFP}}=\mathrm{diag}\left(G_{x},G_{y},G_{z}\right),\;G_{x}=G_{z}=-\frac{G}{2},\;G_{y}=G,$$

and

(51) $$H_{\mathrm{FFP}}=\sqrt{{\left(G_{x}\Delta x\right)}^{2}+{\left(G_{y}\Delta y\right)}^{2}+{\left(G_{z}\Delta z\right)}^{2}}.$$

The fast translation is fixed along *y*, so

(52) $${\hat{v}}_{F}={\hat{e}}_{y}.$$

For FFP-*y* reception, from Equation (23), the collinear component is defined as

(53) $$\begin{array}{cc} h_{\|,y}\left(\Delta r\right)\; & ={\hat{e}}_{y}^{T}h\left(\Delta r\right){\hat{e}}_{y}\\ \; & =A\left(H_{\mathrm{FFP}}\right)\frac{G_{y}^{3}\Delta y^{2}}{H_{\mathrm{FFP}}^{2}}+B\left(H_{\mathrm{FFP}}\right)\left[G_{y}-\frac{G_{y}^{3}\Delta y^{2}}{H_{\mathrm{FFP}}^{2}}\right]. \end{array}$$

Along the fast-scan line, Δx=Δz=0, this reduces to

(54) $$h_{\|,y}\left(\Delta y\right)=G_{y}L^{\prime }\;\left(\beta |G_{y}\Delta y|\right),$$

which is a single-peaked collinear response centered at the FFP.

For FFP-*x* reception, while the scan remains along the y-axis as described in Equation (22), the measured component is transverse

(55) $$h_{⊥,xy}\left(\Delta r\right)={\hat{e}}_{x}^{T}h\left(\Delta r\right){\hat{e}}_{y}.$$

Expanding Equation (49) gives

(56) $$h_{⊥,xy}\left(\Delta x,\Delta y,\Delta z\right)=\left[L^{\prime }\left(\beta H_{\mathrm{FFP}}\right)-\frac{L(\beta H_{\mathrm{FFP}})}{\beta H_{\mathrm{FFP}}}\right]\frac{G_{x}G_{y}^{2}\Delta x\Delta y}{H_{\mathrm{FFP}}^{2}}.$$

With $G_{x}=-G/2$ and $G_{y}=G$,

(57) $$h_{⊥,xy}=-\frac{G^{3}\Delta x\Delta y}{2H_{\mathrm{FFP}}^{2}}\left[L^{\prime }\left(\beta H_{\mathrm{FFP}}\right)-\frac{L(\beta H_{\mathrm{FFP}})}{\beta H_{\mathrm{FFP}}}\right].$$

The transverse component, therefore, satisfies

(58) $$h_{⊥,xy}\left(0,\Delta y,\Delta z\right)=0,\;h_{⊥,xy}\left(\Delta x,0,\Delta z\right)=0.$$

Hence the signed *x*–*y* transverse PSF has alternating lobes separated by nodal lines at Δx=0 and Δy=0. An amplitude-envelope image displays the magnitude $|h_{⊥,xy}|$, so the lobe signs are removed but the central null and multi-lobe structure remain. This behavior is qualitatively different from that of a diagonal $h_{xx}$ component, which produces a single-peaked response.

#### 3.3.2. FFL Matrix-PSF Components

For the FFL configuration,

(59) $$G_{\mathrm{FFL}}=\mathrm{diag}\left(0,G_{y},G_{z}\right),\;G_{y}=-G_{z},$$

and the corresponding field magnitude is

(60) $$H_{\mathrm{FFL}}=\sqrt{{\left(G_{y}\Delta y\right)}^{2}+{\left(G_{z}\Delta z\right)}^{2}}.$$

Since the response is independent of *x*, spatial localization along the FFL direction is intrinsically undefined. For the common *y*-directed translation and a *y*-directed receiver,

(61) $$\begin{array}{cc} h_{\mathrm{FFL},yy}\; & ={\hat{e}}_{y}^{T}h_{\mathrm{FFL}}{\hat{e}}_{y}\\ \; & =A\left(H_{\mathrm{FFL}}\right)\frac{G_{y}^{3}\Delta y^{2}}{H_{\mathrm{FFL}}^{2}}+B\left(H_{\mathrm{FFL}}\right)\left[G_{y}-\frac{G_{y}^{3}\Delta y^{2}}{H_{\mathrm{FFL}}^{2}}\right]. \end{array}$$

This is the encoded collinear response in the transverse *y*–*z* plane.

For the common *y*-directed translation and an *x*-directed receiver,

(62) $$h_{\mathrm{FFL},xy}={\hat{e}}_{x}^{T}h_{\mathrm{FFL}}{\hat{e}}_{y}.$$

Because $G_{x}=0$ and the first component of $G_{\mathrm{FFL}}\Delta r$ is identically zero,

(63) $$h_{\mathrm{FFL},xy}\left(\Delta r\right)=0.$$

Therefore, a nonzero FFL-*x* PSF and associated FWHM cannot be retained under the assumption that AM-MPI and x-space MPI share the same spatial matrix PSF. A nonzero transverse matrix-PSF comparison for a *y*-directed FFL scan would require a receive direction within the encoded plane, for example,

(64) $$h_{\mathrm{FFL},zy}={\hat{e}}_{z}^{T}h_{\mathrm{FFL}}{\hat{e}}_{y}=\left(A-B\right)\frac{G_{z}G_{y}^{2}\Delta z\Delta y}{H_{\mathrm{FFL}}^{2}}.$$

#### 3.3.3. Spatial-Width Metrics

For a normalized, single-peaked PSF profile $\left(|S_{max}|\right)$, the half-maximum locations satisfy,

(65) $$|S_{1}\left|=\right|S_{2}|=\frac{|S_{max}|}{2},$$

and

(66) $$\mathrm{FWHM}=|S_{2}-S_{1}|.$$

**Figure 4:**
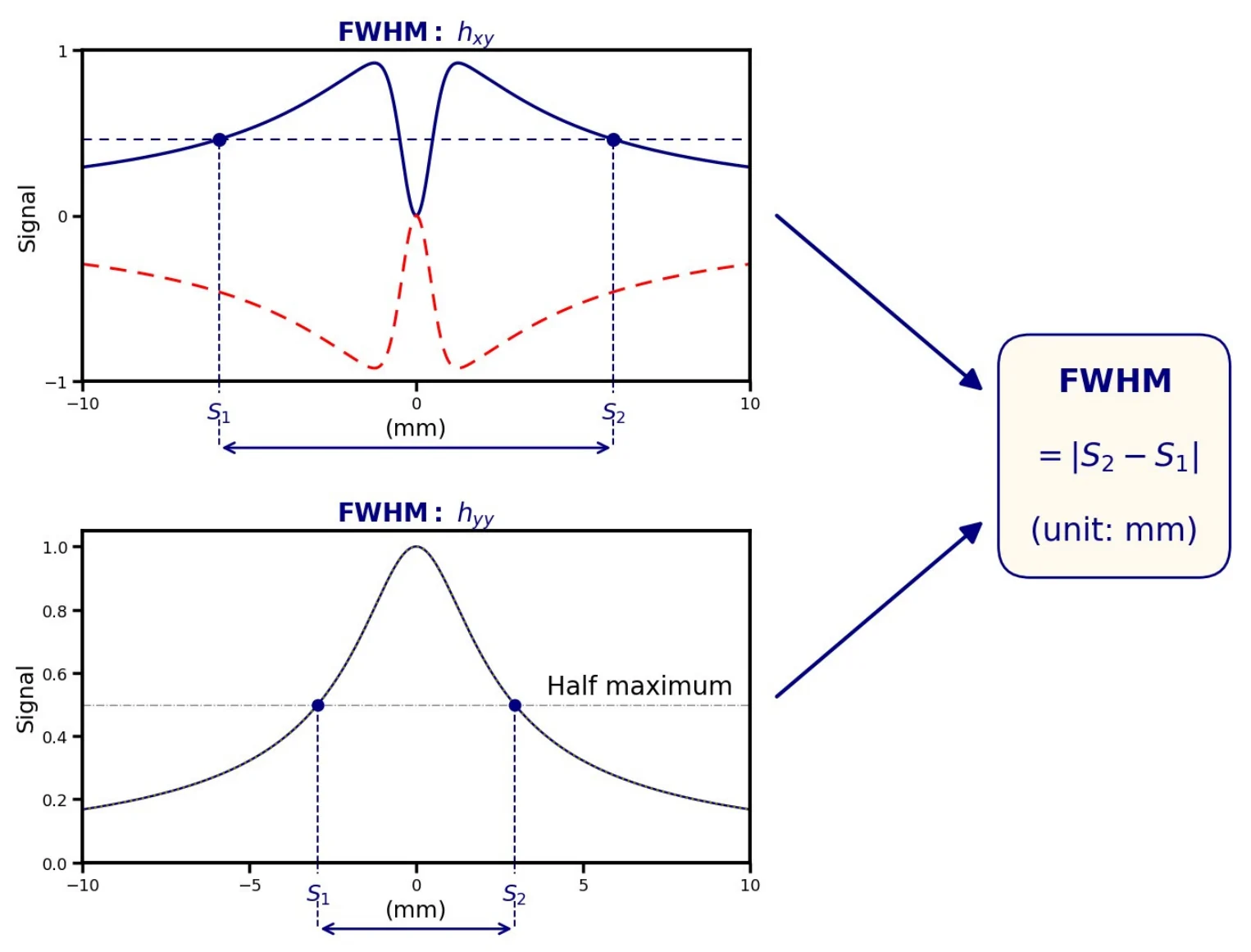
Definition of spatial resolution based on the FWHM of the normalized PSF response.

Figure 4 illustrates the definition of FWHM.

A conventional principal-axis FWHM is not defined for the transverse FFP-*x* component in Equation (56), because the response vanishes on both principal axes through the origin and consists of multiple off-axis lobes. In this analysis, this component is evaluated along the declared diagonal cut satisfying $|G_{x}\Delta x|=|G_{y}\Delta y|$. Two complementary half-maximum metrics are reported: (i) the outer-to-outer half-maximum span of the complete two-lobe magnitude profile, and (ii) the conventional FWHM of an individual lobe. The lobe peak-to-peak separation is reported as an additional topology metric. These quantities characterize the transverse response but are not interpreted as a conventional principal-axis FWHM. The ideal FFL-*x* channel in Equation (63) remains identically zero and, therefore, has no FWHM.

The spatial resolution for different conditions was investigated, as described in detail in the subsections below. The saturation magnetization, volume-weighted mean magnetic core diameter, and the magnetic-field parameters are listed in Table 1.

**Figure 5:**
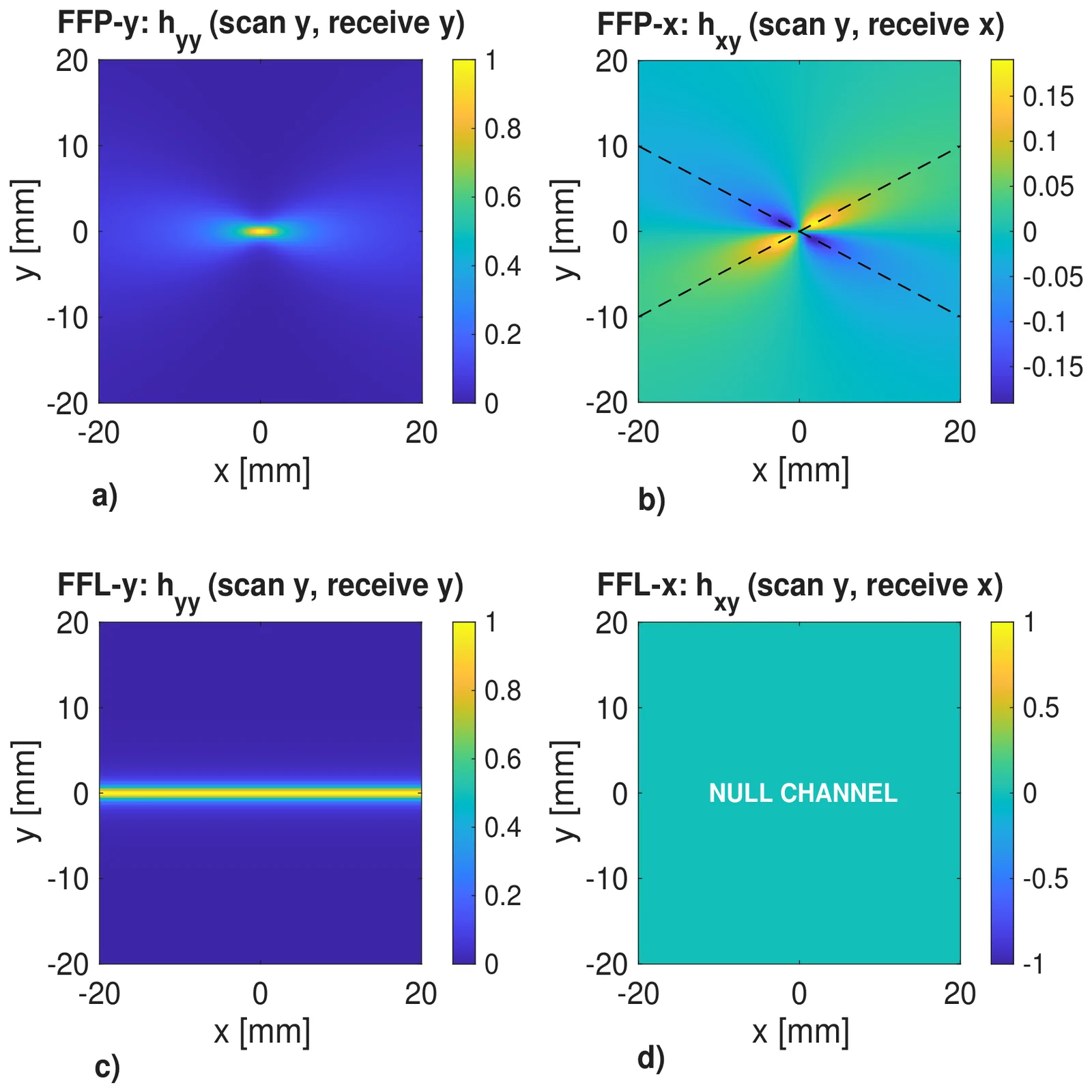
Simulated matrix-PSF distributions for the four investigated FFP and FFL scan–receive configurations in the *x*–*y* plane. (**a**) FFP-*y*: collinear $h_{yy}$ response for *y*-directed scanning and reception. (**b**) FFP-*x*: transverse $h_{xy}$ response for *y*-directed scanning and *x*-directed reception, showing alternating lobes and a central null. (**c**) FFL-*y*: collinear $h_{yy}$ response; the horizontal ridge reflects the intrinsic nonlocality along the *x*-directed FFL. (**d**) FFL-*x*: ideal null $h_{xy}$ channel for the adopted $G_{\mathrm{FFL}}=\mathrm{diag}\left(0,G,-G\right)$ geometry.

**Figure 6:**
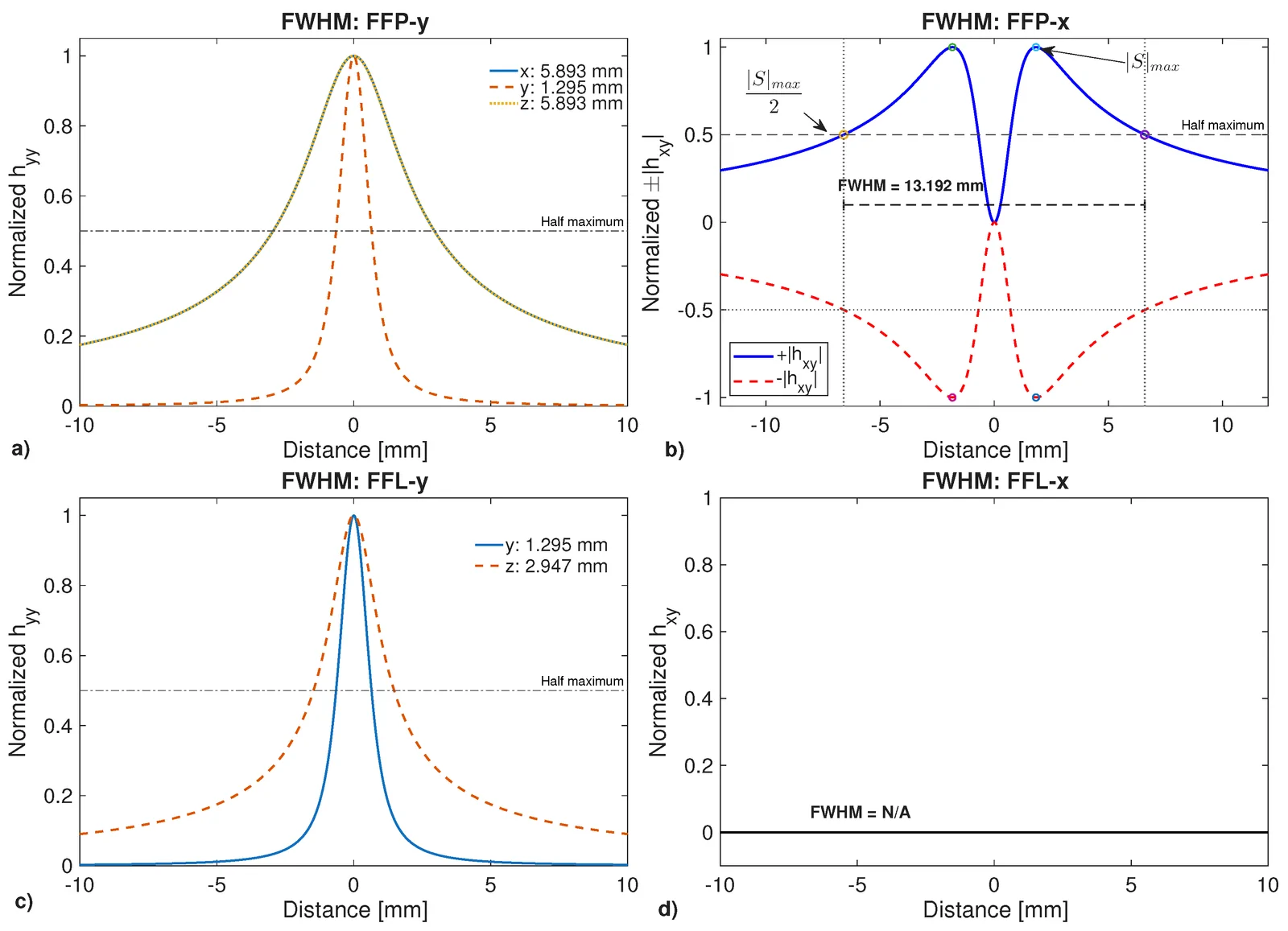
PSF-domain spatial-width metrics for the four investigated FFP and FFL scan–receive configurations. (**a**) FFP-*y* collinear $h_{yy}$ response. (**b**) FFP-*x* transverse $h_{xy}$ response. (**c**) FFL-*y* encoded-plane FWHM values. (**d**) FFL-*x* is an ideal null channel.

Figure 5 shows the PSF obtained from the matrix-PSF projection for the four scan–receive configurations. For FFP-*y*, the *y*-directed scan and *y*-directed receiver select the collinear $h_{yy}$ component, producing a single localized peak centered at the FFP. For FFP-*x*, the same *y*-directed scan with an *x*-directed receiver selects the transverse $h_{xy}$ component. The resulting signed PSF exhibits alternating off-axis lobes separated by nodal lines at Δx=0 and Δy=0, and its magnitude retains the characteristic central null. The dashed diagonal cuts in Figure 5 pass through the transverse lobes and are used for the component-specific width analysis in Figure 6.

The FFL scans in Figure 5 also make the localization geometry explicit. Because $G_{x}=0$, the FFL extends along the *x*-direction. Accordingly, FFL-*y* appears as a horizontal *x*-directed ridge in the *x*–*y* plane rather than as a point-localized response. Its finite resolution is defined only in the encoded *y*–*z* plane. In contrast, FFL-*x* remains an ideal null channel because the *x*-directed receive axis selects $h_{xy}=0$ for the adopted *y*-directed translation.

Figure 6 quantifies the PSF-domain spatial widths. FFP-*y* is single-peaked and yields conventional FWHM values of 5.89mm along *x*, 1.29mm along *y*, and 5.89mm along *z*. For FFP-*x*, a conventional principal-axis FWHM is not defined. Along the specified diagonal cut, the FWHM = 13.192mm. FFL-*y* yields encoded-plane FWHM values of 1.29mm along *y* and 2.94mm along *z*, while the *x*-direction remains undefined. FFL-*x* is identically zero and, therefore, has no FWHM. Table 2 summarizes these component-appropriate metrics.

The four-case comparison presented in Figure 5 and Figure 6 consistently reflects the distinct spatial-response characteristics of the four configurations: the FFP-*y* collinear response, the FFP-*x* transverse multi-lobe response, the nonlocal FFL-*y* ridge response, and the ideal FFL-*x* null response. Accordingly, the spatial-width values used in the subsequent analysis are those in Table 2.

## 4. Phantom Scanning Simulation

### 4.1. Phantom Simulation and Image Reconstruction

The phantom reconstruction simulation aims to determine whether the spatial-resolution patterns predicted by the PSF and FWHM analyses are observable in the reconstructed image domain of AM-MPI. The objective is not only to present the reconstructed images but also to determine whether a narrower PSF produces a more localized reconstructed response and whether an undefined FWHM along a given direction leads to line-integrated or slice-ambiguous reconstruction.

The numerical phantom is discretized into an image grid with $\left(N_{x}\times N_{y}\times N_{z}\right)$ pixels. The unknown MNPs concentration map is written in vector form as

(67) $$c={\left[c_{1},c_{2},\ldots ,c_{N}\right]}^{T},\;N=N_{x}N_{y}N_{z},$$

where $c_{i}$ denotes the particle concentration at the *i*-th pixel. During scanning, each time sample $\left(t_{k}\right)$ corresponds to a spatial location of the FFP or FFL inside the field of view.

The AM-MPI image is reconstructed by assigning the extracted signal amplitude to the corresponding FFP/FFL trajectory. For the FFP case, the reconstructed image can be written as

(68) $$IMG_{q}\left(r_{\mathrm{FFP}\text{-}x,\;\mathrm{FFP}\text{-}y}\left(t\right)\right)=Amp\left[u^{PR}\left(r_{\mathrm{FFP}\text{-}x,\;\mathrm{FFP}\text{-}y}\left(t\right)\right)\right].$$

For the FFL case, the extracted amplitude corresponds to a line-integrated response along the field-free line direction and is assigned to the transverse FFL position:

(69) $$IMG_{q}\left(r_{\mathrm{FFL}\text{-}y,\;\mathrm{FFL}\text{-}x}\left(t\right)\right)=Amp\left[u_{q}^{PR}\left(r_{\mathrm{FFL}\text{-}y,\;\mathrm{FFL}\text{-}x}\left(t\right)\right)\right].$$

The discrete image is then obtained by interpolating the irregularly sampled amplitude values onto a Cartesian image grid.

It is important to distinguish the PSF-domain FWHM from a reconstructed-image FWHM. In this work, the PSF-domain FWHM is defined directly from the normalized orientation-dependent point-spread function and is used as the intrinsic spatial-resolution metric. A separate reconstructed-image FWHM is not reported in the present analysis. This is because the reconstruction does not apply an independent deconvolution or system-matrix inversion that would produce a distinct image-domain impulse response. Instead, the reconstructed image is formed by assigning the demodulated carrier-envelope amplitudes to the corresponding FFP/FFL scan locations and subsequently interpolating these samples onto a Cartesian grid. Consequently, the apparent width of a reconstructed spot also depends on the discrete raster sampling and interpolation grid and, therefore, should not be interpreted as an independent measure of the intrinsic FWHM of the AM-MPI system.

For this reason, all FWHM values reported in Table 2 and used to normalize the two-point source spacing are explicitly PSF-domain values, denoted by ${\mathrm{FWHM}}_{\mathrm{PSF}}$. The reconstructed numerical phantoms are used separately to assess image-domain two-point separability through the one-dimensional line profiles and the modulation-depth metric defined below. This separation prevents interpolation-dependent reconstructed spot widths from being conflated with the intrinsic PSF-domain resolution.

The phantom analysis must use a spatial metric compatible with the selected matrix-PSF component. For the single-peaked FFP-*y* and FFL-*y* collinear channels, source separation can be normalized by the corresponding directional PSF-domain FWHM. For the transverse FFP-*x* channel, the PSF has no conventional principal-axis FWHM; therefore, any normalized two-point comparison must explicitly state the applied transverse metric. The present PSF analysis provides an overall diagonal half-maximum span of 13.192mm, an individual-lobe FWHM of 5.896mm, and a lobe peak-to-peak separation of 3.663mm. The ideal FFL-*x* channel is zero and is excluded from two-point separability analysis under the strict matrix-PSF model.

To quantify two-point separability objectively, the one-dimensional reconstructed intensity profile along the source-separation direction was additionally evaluated using the modulation depth. Let $P_{1}$ and $P_{2}$ denote the intensities of the two local maxima and *V* the local minimum between them. The modulation depth is calculated as

(70) $$M=1-\frac{V}{\frac{P_{1}+P_{2}}{2}},$$

where a larger *M* indicates a deeper valley between the two reconstructed responses and, therefore, stronger two-point separability. Following established Sparrow and Rayleigh two-point resolution criteria, the emergence of two local maxima separated by a local minimum (M>0) was taken as the onset of resolvability, whereas $M\geq 0.26(\frac{V}{P}\leq 0.74)$ was used as a more conservative Rayleigh-type reference for well-resolved sources [32]. For single-peaked collinear channels, source separation is normalized by the corresponding directional PSF FWHM. For transverse multi-lobe channels, a component-specific lobe metric must be stated explicitly instead of a central FWHM. These criteria are used as reference measures rather than universal MPI resolution limits.

To ensure a fair comparison, the investigated configurations were evaluated using consistent spatial sampling, excitation conditions, and interpolation settings. The excitation field was fixed at $H_{E}=2\;\mathrm{mT}/\mu_{0}$ and $f_{E}=20\;\mathrm{kHz}$. For the FFP geometry, the signed gradient components were $G_{y}=2.5\;T\;m^{-1}/\mu_{0}$ and $G_{x}=G_{z}=-1.25\;T\;m^{-1}/\mu_{0}$. For the FFL geometry, the signed components were $G_{x}=0$, $G_{y}=2.5\;T\;m^{-1}/\mu_{0}$, and $G_{z}=-2.5\;T\;m^{-1}/\mu_{0}$. The FFP was sampled using the line-by-line trajectory defined in Equation (20), while the FFL was translated only in the transverse *y*-*z* plane according to Equation (25); no *x*-directed drive field was applied in the FFL simulations.

The phantom simulations are spatial-response calculations and do not explicitly time-discretize the raster trajectory. Therefore, no temporal sampling frequency, fast-line repetition rate, samples-per-line value, or frame duration is imposed in the present PSF/phantom code. The directional single-lobe profiles used to determine the local FWHM values were evaluated over [−20,20]mm using 80,001 samples, corresponding to $5\times 10^{-4}\;\mathrm{mm}$. Each two-dimensional phantom/reconstruction panel used a 401×301 Cartesian grid over a direction-dependent symmetric field of view that contained both sources and their PSF extent.

### 4.2. Absolute-Separation Simulation

The absolute-separation analysis asks a practical question: how well can each configuration distinguish two sources placed at the same physical distance? We, therefore, used (d=[1.0,3.0,5.0,7.0]mm) for all investigated FFP/FFL configurations and separation directions, without scaling the source spacing by the corresponding directional PSF width.

Measurement noise was omitted so that the comparison isolates the deterministic effects of field geometry and excitation–receive orientation. The results, therefore, represent ideal PSF and reconstruction behavior under matched numerical conditions rather than experimentally achievable resolution.

**Figure 7:**
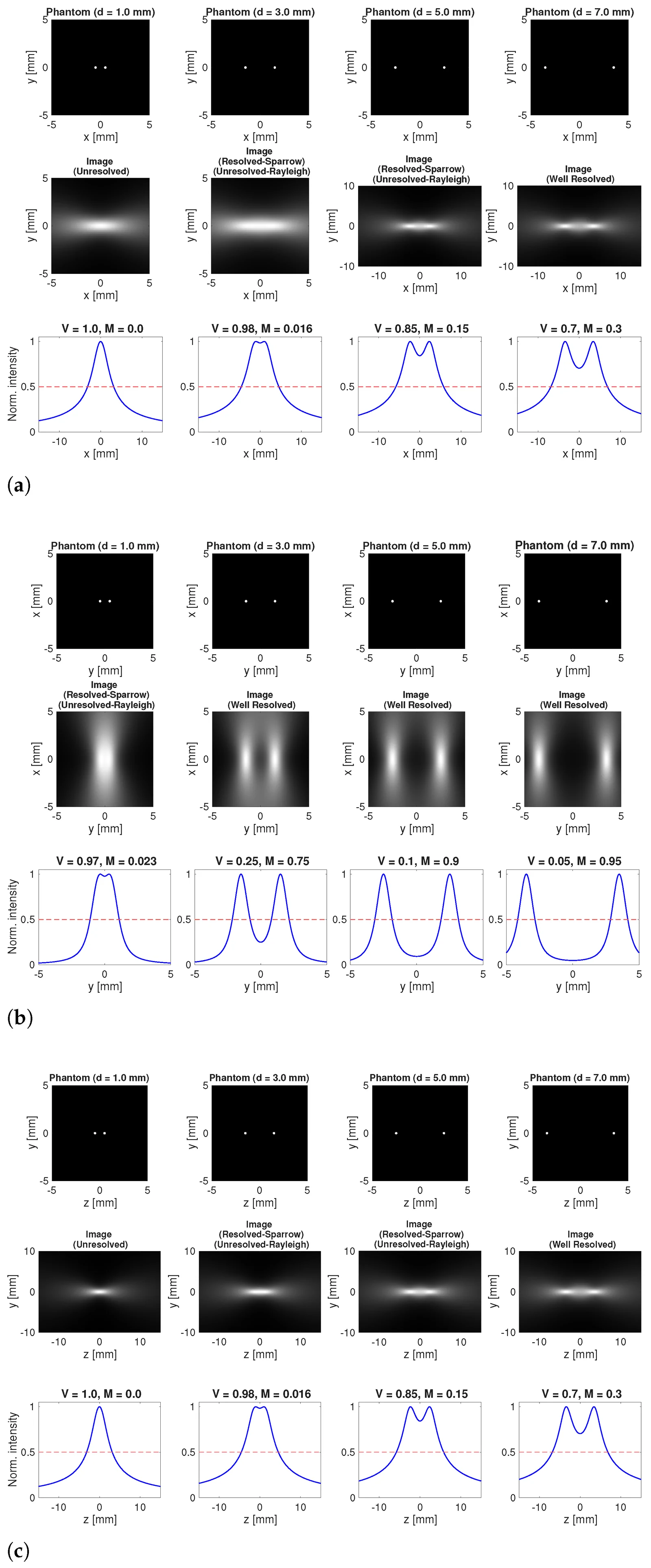
Two-point separability of the FFP-*y* configuration at equal absolute source separations *d*: separation along (**a**) *x*-direction, (**b**) *y*-direction, and (**c**) *z*-direction.

Figure 7 shows that FFP-*y* resolves equal physical separations most clearly along *y*. The identical *x*- and *z*-direction responses follow from the gradient symmetry $G_{x}=G_{z}=-G/2$. Thus, the stronger encoding $|G_{y}|=G$ gives FFP-*y* its best absolute two-point localization along *y*.

**Figure 8:**
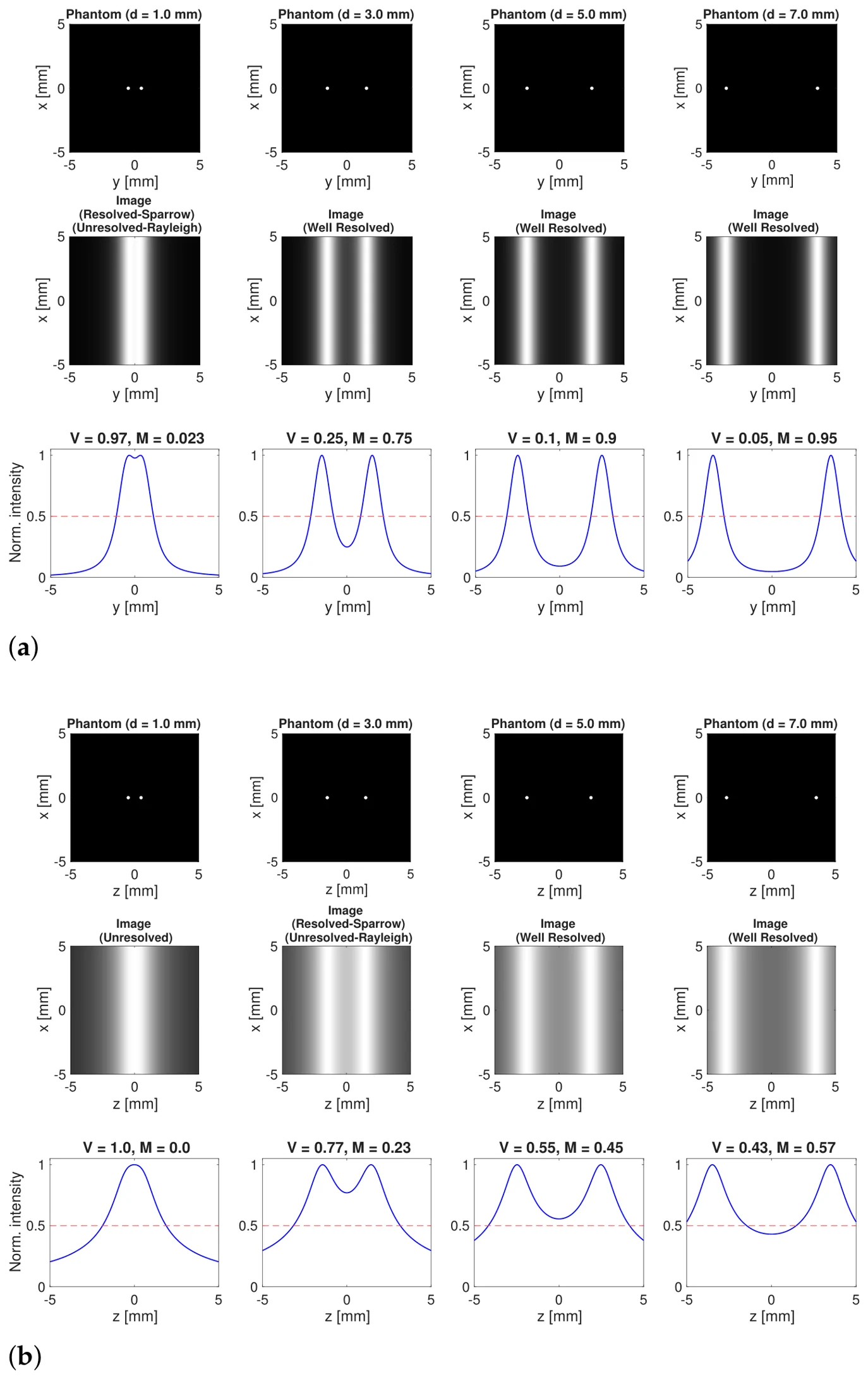
Two-point separability of the FFL-*y* configuration at equal absolute source separations *d*: separation along (**a**) *y*-direction and (**b**) *z*-direction.

Figure 8 shows that FFL-*y* matches FFP-*y* along *y*: both use $|G_{y}|=G$ encoding and have a directional FWHM of 1.29mm. However, along z, FFL-y is narrower than FFP-y, with FWHM values of 2.94 and 5.89 mm, respectively. FFL-*y*, therefore, provides better *z*-direction separability at the same physical spacing. This improvement is transverse only, because each reconstructed source remains extended along the FFL direction *x*. The best performance is obtained by FFP-*y* and FFL-*y* along *y*, followed by FFL-*y* along *z*. The FFP-*y* along *x* or *z* requires the largest physical separation. FFP-*x* is excluded from this width-based ranking because its nodal valleys strongly influence its modulation depth.

### 4.3. FWHM-Normalized Separation Simulation

To compare two-point separability relative to the directional PSF width, the source spacing was normalized as $d_{\mathrm{norm}}=d/{\mathrm{FWHM}}_{\alpha }$, where ${\mathrm{FWHM}}_{\alpha }$ is the PSF-domain FWHM in separation direction α with value $d_{\mathrm{norm}}=\left[0.5,1.0,1.5,2.0\right]$ and used $d=d_{\mathrm{norm}}{\mathrm{FWHM}}_{\alpha }$ to obtain the corresponding physical spacing. The conventional width-normalized comparison focuses on the single-peaked FFP-*y* and FFL-*y* responses. The transverse FFP-*x* values are retained only as selected-lobe topology controls, whereas the ideal FFL-*x* null channel is excluded.

**Figure 9:**
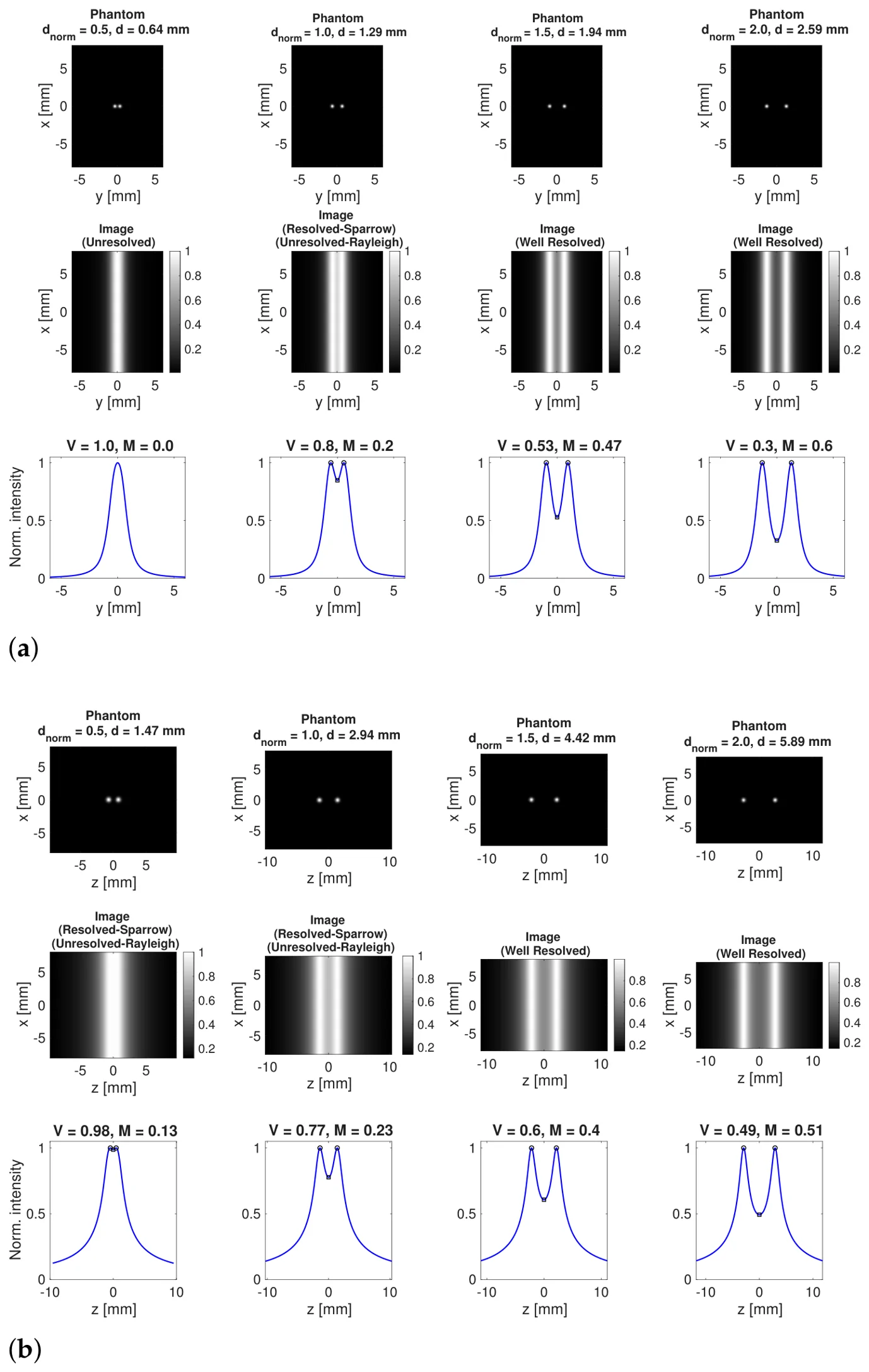
Two-point separability of the FFL-*y* configuration at equal FWHM-normalized source separations $d_{\mathrm{norm}}$: separation along (**a**) *y*-direction and (**b**) *z*-direction.

Figure 9 gives the corresponding FFL-*y* results. Its *y*-direction response is identical to that of FFP-*y* because both share the $h_{yy}$ component and a directional FWHM of 1.29mm. At $d_{\mathrm{norm}}=1.5$, the required physical spacing is 1.942mm along *y* and 4.421mm along *z*. The latter is half the 8.839mm required by FFP-*y* along *z*, demonstrating the benefit of the larger $|G_{z}|$ in the FFL selection-gradient tensor. The improvement remains transverse because FFL-*y* is nonlocal along *x*.

**Figure 10:**
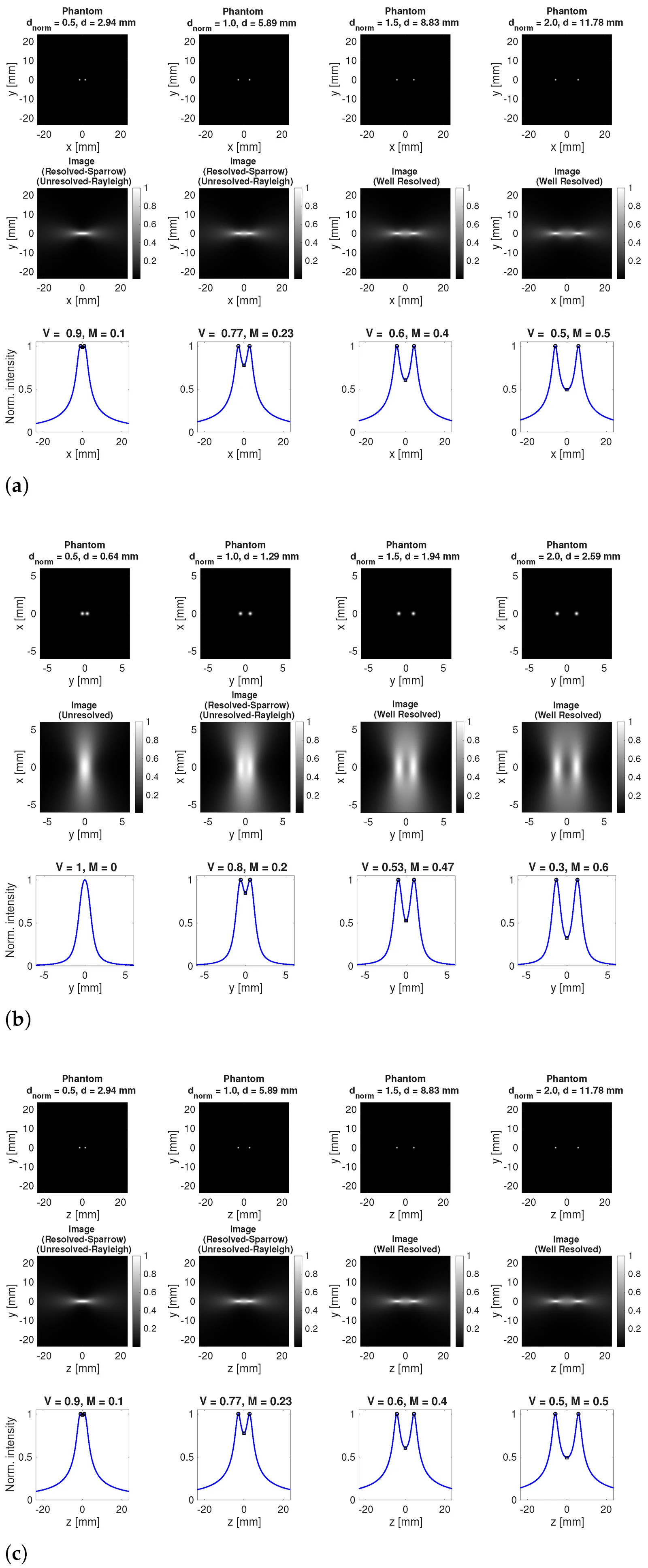
Two-point separability of the FFP-*y* configuration at equal FWHM-normalized source separations $d_{\mathrm{norm}}$: separation along (**a**) *x*-direction, (**b**) *y*-direction, and (**c**) *z*-direction.

Figure 10 shows the expected transition from one merged response to two distinguishable maxima as $d_{\mathrm{norm}}$ increases. The adopted Rayleigh-type criterion M≥0.26 is reached at approximately $d_{\mathrm{norm}}=1.5$ in all three directions. The corresponding physical spacing is 1.942mm along *y* but 8.839mm along both *x* and *z*. Thus, normalization produces comparable relative overlap, while the physical distances retain the practical advantage of the narrower *y*-direction PSF.

For equal absolute source spacing, the modulation depth strongly depends on the directional PSF width. The FFP-y and FFL-y configurations exhibit identical performance along the *y*-direction because both have the same directional FWHM of 1.29mm. At d=1mm, the two sources already exhibit a shallow valley with M=0.023, satisfying the adopted Sparrow-type onset criterion but remaining below the Rayleigh-type reference. At d=3mm, the modulation depth increases to M=0.75, making this the first tested absolute separation that satisfies the adopted M≥0.26 Rayleigh-type criterion along *y*. The modulation depth further increases to 0.9 and 0.95 at d=5 and 7mm, respectively.

In contrast, the broader FFP-y responses along *x* and *z*, both with FWHM =5.89mm, remain below the Rayleigh-type criterion at d=3 and 5mm, with M=0.016 and 0.15, respectively. The criterion is first satisfied among the tested spacings at d=7mm, where M=0.3. FFL-y shows better absolute separability along *z* because of its narrower 2.94mm directional FWHM. Its modulation depth increases from M=0 at d=1mm to 0.23 at 3mm, and reaches 0.45 at 5mm, which is the first tested spacing that satisfies the Rayleigh-type criterion. These results directly demonstrate the practical resolution advantage of the stronger encoded-gradient directions. The modulation-depth results for both equal absolute and FWHM-normalized source separations are summarized in Table 3.

### 4.4. Discussion

Field orientation affects not only the directional spatial width of the AM-MPI response but also the shape of the measured matrix-PSF component. With the same y-directed scan, changing the receive axis from y to x changes the measured matrix-PSF component from the collinear $h_{yy}$ response to the transverse $h_{xy}$ response. The former has a single central peak and can be described by a conventional FWHM, whereas the latter contains a central null and multiple off-axis lobes. The FFL cases have a different spatial character. FFL-y remains spatially encoded in the y-z plane but is nonlocal along x, while FFL-x is a null channel for the field geometry considered here. A single resolution measure, therefore, cannot be applied to all four configurations. FWHM is appropriate for the single-peaked FFP-y and FFL-y responses, whereas the FFP-x response requires lobe-specific measures and FFL-x has no meaningful spatial width under the adopted model. The difference between FFP-y and FFL-y is closely related to their selection-field tensors. For the FFP, $G_{y}=G$ and $G_{x}=G_{z}=-G/2$, giving a FWHM of 1.29 mm along y and 5.89 mm along x and z. For the FFL, $G_{y}=G$ and $G_{z}=-G$. The y-direction FWHM, therefore, remains 1.29 mm, while the z-direction FWHM decreases to 2.94 mm. The better z-direction performance of FFL-y should not be interpreted simply as a general advantage of FFL encoding. It follows from the larger encoded $|G_{z}|$ and is accompanied by the loss of intrinsic localization along x because $G_{x}=0$. FFP and FFL, therefore, provide different spatial trade-offs: FFP supports point localization in three dimensions, whereas FFL provides stronger localization within the encoded transverse plane but retains ambiguity along the field-free line. The two-point simulations show how these differences appear at fixed physical source spacing. Along y, FFP-y and FFL-y behave identically because they have the same directional PSF width. At d=1mm, both yield M=0.023, indicating the onset of two-peak separation according to the adopted Sparrow-type criterion while remaining below the Rayleigh-type reference. At d=3mm, the modulation depth increases to M=0.75, making this the first tested absolute spacing that satisfies the Rayleigh-type criterion.

The broader FFP-y response along *x* and *z* requires substantially larger physical spacing: *M* increases from 0 at 1mm to 0.016, 0.15, and 0.3 at 3, 5, and 7mm, respectively. Thus, 7mm is the first tested spacing that satisfies the Rayleigh-type criterion in these directions. FFL-y provides improved *z*-direction separability, reaching M=0.23 at 3mm and M=0.45 at 5mm; therefore, 5mm is the first tested spacing satisfying the Rayleigh-type criterion along *z*. The absolute-separation analysis, therefore, directly demonstrates how the directional PSF width determines the physical distance required for reliable two-source separation.

**Figure 11:**
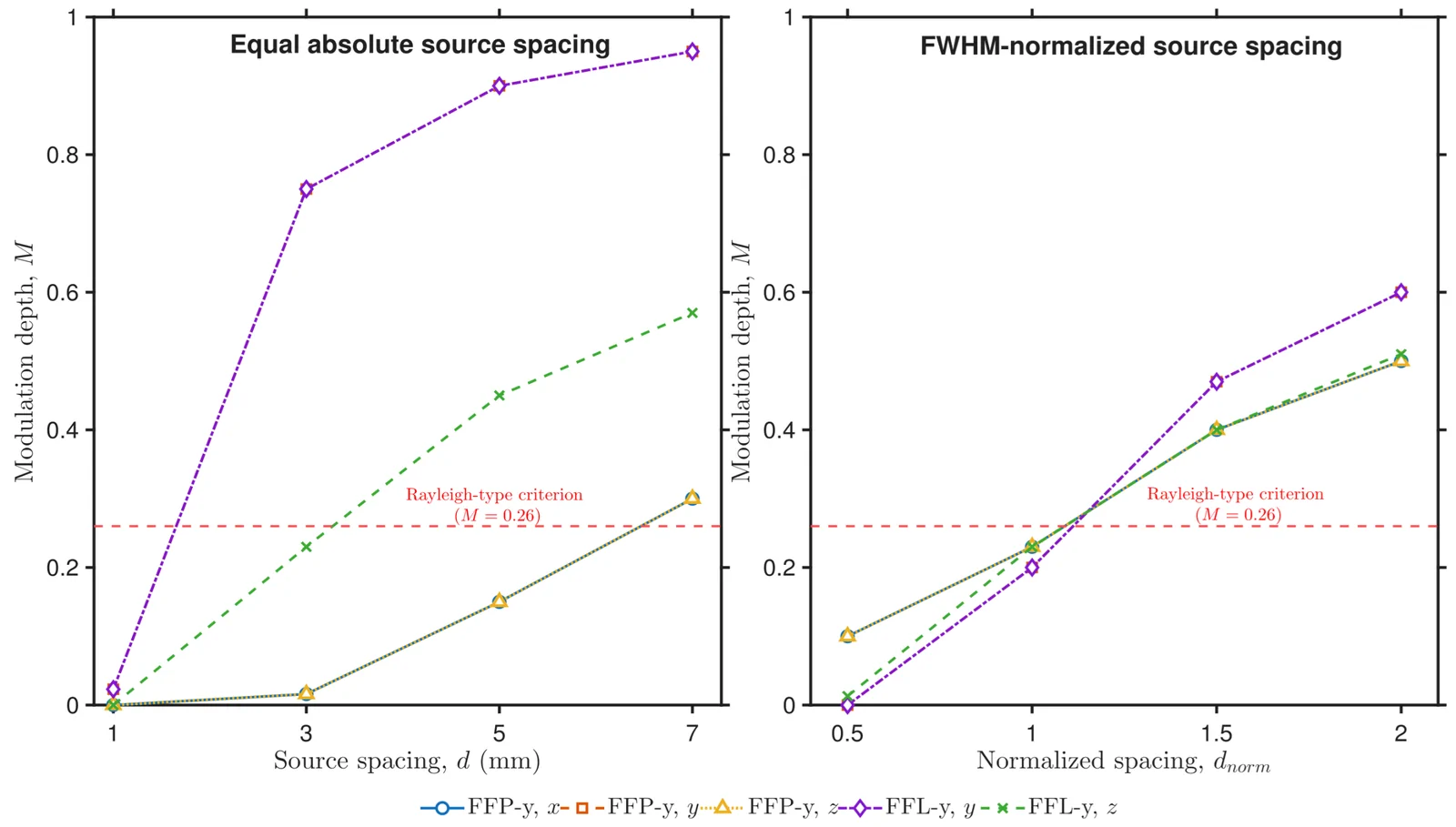
Modulation-depth comparison for equal absolute and FWHM-normalized source separations. The dashed red line indicates the adopted Rayleigh-type reference criterion (M = 0.26).

Normalization by the directional FWHM gives a complementary view. At the same $d_{norm}$, the single-peaked responses become much more similar. At $d_{norm}$ = 1.5, the FFP-y (x- and z-direction) responses and the FFL-y z-direction response give M≈0.4, whereas the common y-direction response gives M≈0.47. Much of the difference observed at equal physical spacing is, therefore, associated with directional PSF width rather than with a different basic mechanism of two-source separation. The remaining difference is consistent with the different shapes of the corresponding one-dimensional cuts through $h_{yy}$. Absolute and normalized analyses in Figure 11 answer different questions: the former describes separation in millimeters, whereas the latter compares source overlap relative to the width of the corresponding PSF. The interpretation of modulation depth depends on the resolution criterion being used. In this work, M>0 corresponds to the onset of two-peak separation according to the adopted Sparrow-type criterion, whereas the Rayleigh-type criterion requires M≥0.26. A case with 0<M<0.26 can, therefore, satisfy the Sparrow criterion while remaining unresolved according to the Rayleigh criterion. This distinction should be stated explicitly when the reconstructed images are classified. The normalized simulations were performed only at $d_{norm}$ = 0.5, 1.0, 1.5, and 2.0. Accordingly, $d_{norm}$ = 1.5 is the first tested normalized separation that satisfies the Rayleigh-type criterion and should not be interpreted as the exact threshold. A finer spacing interval would be required to determine the crossing point more precisely. Resolution alone is not sufficient for selecting a field orientation. Collinear and transverse matrix-PSF components can differ substantially in signal amplitude, which becomes important once measurement noise and residual excitation feedthrough are considered [30]. The present simulations were designed to isolate spatial-response effects and do not include an amplitude- or SNR-based comparison. In practice, a narrow or otherwise favorable spatial response may provide limited benefit if the corresponding receive signal is weak. Future optimization should, therefore, examine spatial resolution together with signal strength, SNR, and the receive-coil configuration rather than treating these quantities independently. The present study uses one common fast-scan direction and a single receive direction for each configuration. These choices make the orientation effects easier to isolate, but they do not cover the full set of encoding options available in a three-dimensional AM-MPI system. Future work should examine how changing the scan direction alters the selected matrix-PSF component and whether signals acquired from receive coils oriented along multiple axes can provide complementary spatial information. Multi-axis reception may be particularly useful when one receive direction produces a weak or topologically unfavorable response. The orientation of the FFL itself is another variable that deserves separate study. Rotating the FFL would change the direction of the nonlocal axis and the orientation of the encoded transverse plane; measurements obtained at several FFL orientations could provide complementary projections and may help reduce the localization ambiguity that is intrinsic to a single fixed FFL. These extensions would allow scan direction, receive direction, and field-free-region orientation to be considered jointly rather than as independent hardware choices. The reported values represent idealized spatial-response behavior. The particle model assumes an equilibrium monodisperse Langevin response and does not include Brownian or Néel relaxation, dynamic phase lag, or particle-size distributions. Receive sensitivity is treated as spatially uniform, excitation cancellation is idealized, and measurement noise is omitted. The phantom simulations also do not explicitly discretize the raster trajectory in time. These assumptions are useful for separating the effects of field geometry and orientation, but they mean that the reported FWHM and modulation-depth values should not be taken as direct predictions of experimental resolution. Experimental measurements will be needed to determine how tracer dynamics, noise, field nonuniformity, coil misalignment, finite sampling, and multi-axis receive sensitivity modify the orientation-dependent responses described here. For navigation-oriented AM-MPI, the scan direction, receive axis, and selection-field geometry should be chosen together. Aligning an encoded direction with a stronger selection gradient can reduce the corresponding spatial width, but the topology of the selected response and the localization properties of the field-free geometry remain equally important. The most suitable configuration will depend on whether the application requires full three-dimensional localization or places greater weight on resolution within a particular direction or plane. A broader comparison that includes multiple receive axes, alternative scan directions, and rotated FFL orientations is, therefore, a natural next step toward a more complete three-dimensional encoding strategy.

## 5. Conclusions

This study shows that field orientation in AM-MPI affects both the topology and directional width of the spatial response. With a common y-directed scan, FFP-y and FFL-y produce single-peaked collinear responses with the same 1.29-mm FWHM along y, while FFL-y reduces the z-direction FWHM from 5.89 to 2.94 mm because of its stronger transverse encoding. This improvement is accompanied by the intrinsic loss of localization along the FFL direction. In contrast, FFP-x produces a transverse multi-lobe response with a central null, whereas FFL-x is a null channel for the adopted geometry. These differences show that FFP and FFL configurations cannot be compared using a single conventional FWHM measure. The two-point simulations further show that absolute source separability is governed strongly by directional PSF width. Among the tested absolute spacings, FFP-y and FFL-y first satisfy the adopted Rayleigh-type criterion at d=3mm along *y*, while FFL-y reaches the criterion at 5mm along *z* and the broader FFP-y response requires 7mm along *x* and *z*. After normalization by the corresponding FWHM, the single-peaked responses show similar separability, indicating that much of the difference in absolute resolution arises from directional PSF scaling. Field orientation and selection-field geometry should, therefore, be considered together when designing AM-MPI systems for navigation. Future work will extend the analysis to alternative scan directions, multi-axis receive configurations, rotated FFL orientations, signal-to-noise effects, and experimental validation under realistic tracer and hardware conditions.

## Figures and Tables

**Table 1:** Representative iron-oxide nanoparticle parameters used for PSF and spatial-resolution simulations.

Category	Parameter	Value	Unit
Particles	$d_{p}$	30	nm
	$M_{s}$	374,000	A/m
	ρ	5170	$\mathrm{kg}/m^{3}$
	$H_{E}$	2	$\mathrm{mT}/\mu_{0}$
AC magnetic field	*f*	20	kHz
Magnetic field gradient	*G*	2.5	$T\;m^{-1}/\mu_{0}$
Temperature	*T*	298	K

$d_{p}$: Representative diameter used in the model, $M_{s}$: Saturation magnetization.

**Table 2:** PSF components and spatial-width metrics obtained from the simulation.

Configuration	Response Type	*y*-FWHM	*x*- and *z*-FWHM
FFP-*x*	$h_{xy}$; transverse	diagonal: 13.192mm; *y*-: N/A	N/A
FFL-*x*	$h_{xy}=0$; null channel	N/A	N/A
FFP-*y*	$h_{yy}$; collinear	*y*-: 1.29mm	*x*- =z-=5.89mm
FFL-*y*	$h_{yy}$; collinear	*y*-: 1.29mm	*x*-: undefined; *z*- =2.94mm

**Table 3:** Comparison of modulation depth for equal absolute and FWHM-normalized source separations.

Configuration	Direction	Absolute Separation				FWHM-Normalized Separation			
		1.0	3.0	5.0	7.0	0.5	1.0	1.5	2.0
FFP-y	*x*	0.0	0.016	0.15	0.3	0.1	0.23	0.4	0.5
FFP-y	*y*	0.023	0.75	0.9	0.95	0.0	0.2	0.47	0.6
FFP-y	*z*	0.0	0.016	0.15	0.3	0.1	0.23	0.4	0.5
FFL-y	*y*	0.023	0.75	0.9	0.95	0.0	0.2	0.47	0.6
FFL-y	*z*	0.0	0.23	0.45	0.57	0.013	0.23	0.4	0.51

For the absolute-separation analysis, the physical source spacing is fixed at d=[1.0,3.0,5.0,7.0] mm. For the normalized analysis, the physical spacing is calculated as $d=d_{\mathrm{norm}}{\mathrm{FWHM}}_{\alpha }$.

## Data Availability

Data are contained within the article.

## Abbreviations

The following abbreviations are used in this manuscript:

MPI	Magnetic particle imaging
MNPs	Magnetic nanoparticles
SPIONs	Superparamagnetic iron oxide nanoparticles
AM-MPI	Amplitude-modulation magnetic particle imaging
FFP	Field-free point
FFL	Field-free line
FFP-*x*-excitation	*y*-directed FFP scan with *x*-directed excitation/receive axis;
	transverse $h_{xy}$ channel
FFP-*y*-excitation	*y*-directed FFP scan with *y*-directed excitation/receive axis;
	collinear $h_{yy}$ channel
FFL-*y*-excitation	*y*-directed FFL scan with encoded *y*-directed receive axis;
	collinear $h_{yy}$ channel
FFL-*x*-excitation	*y*-directed FFL scan with receive axis parallel to the *x*-directed FFL
PSF	Point-spread function
MRI	Magnetic resonance imaging
CT	Computed tomography
PET	Positron emission tomography
US	Ultrasound
